# Targeting Macrophage‐Mediated Lymphatic Immunosuppression to Prevent Tumor Recurrence After Microwave Ablation

**DOI:** 10.1002/advs.78058

**Published:** 2026-09-28

**Authors:** Xuexia Shan, Yuting Shen, Xiaodong Hou, Fengshan Jin, Shaoyue Li, Weichen Xu, Weiwei Ren, Xiaowan Bo, Qiuyan Li, Shuo Liu, Weifan Hao, Chengzhong Peng, Huixiong Xu, Liping Sun, Wenwen Yue

**Affiliations:** ^1^ Department of Medical Ultrasound Center of Minimally Invasive Treatment for Tumor Shanghai Tenth People's Hospital School of Medicine Tongji University Shanghai P. R. China; ^2^ Department of Ultrasound Zhongshan Hospital Institute of Ultrasound in Medicine and Engineering Fudan University Shanghai P. R. China; ^3^ Department of Ultrasound Shenzhen Maternity and Child Healthcare Hospital Women and Children's Medical Center Southern Medical University Shenzhen P. R. China; ^4^ Department of Medical Ultrasound Shanghai Municipal Hospital of Traditional Chinese Medicine Shanghai University of Traditional Chinese Medicine Shanghai P. R. China; ^5^ The First Hospital of Anhui University of Science & Technology Anhui University of Science and Technology Huainan P. R. China

**Keywords:** drug delivery, immune microenvironment, lymph nodes, microwave ablation, tumor immunotherapy

## Abstract

Microwave ablation (MWA) is an effective locoregional therapy for solid tumors, but recurrence caused by minimal residual disease remains a clinical challenge. Although immunotherapy offers a strategy to prevent recurrence, the role of tumor‐draining lymph nodes (TDLNs) in post‐ablation immune regulation remains underexplored. Here, single‐cell RNA sequencing (scRNA‐seq) reveals pronounced macrophage remodeling in TDLNs following incomplete MWA (iMWA), characterized by an increased representation of immunosuppressive *Folr2^+^* and *Apoe^+^* macrophage subsets. These populations exhibit broad colony‐stimulating factor 1 receptor (*Csf1r*) expression, accompanied by enhanced CSF signaling and macrophage‐centered intercellular communication, implicating CSF1R‐associated macrophage remodeling in post‐ablation immunosuppression. Accordingly, an albumin‐based lipoprotein nanosystem co‐encapsulating paclitaxel (PTX) and the CSF1R inhibitor BLZ945 (PTX/BLZ945@Alb‐lip) was developed. Across multiple tumor models, PTX/BLZ945@Alb‐lip enhances immunomodulator delivery to both TDLNs and tumors, remodels the macrophage‐mediated immunosuppressive microenvironment, and suppresses residual tumor growth after iMWA. Moreover, treatment promotes durable antitumor immune memory and protects cured mice against tumor rechallenge. These findings identify TDLNs as an important therapeutic site in post‐ablation immunotherapy and provide a potential strategy to prevent tumor recurrence following ablation.

## Graphical abstract

Incomplete microwave ablation induces animmunosuppressive microenvironment in tumor‐draining lymph nodes. An albumin‐based lipoprotein nanosystem enables coordinated delivery of immunomodulators to tumor‐draining lymph nodes and residual tumors, reverses post‐ablation immunosuppression, suppresses residual tumor growth, and promotes durable antitumor immune memory against tumor rechallenge.

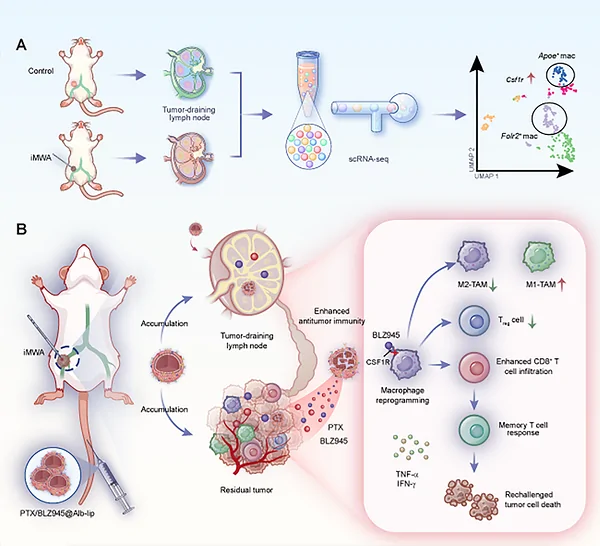

## 1 Introduction

Microwave ablation (MWA), along with other thermal ablation therapies such as high‐intensity focused ultrasound and radiofrequency ablation, has undergone significant advancements over the past two decades [1] and is increasingly regarded as a standard treatment option for various solid tumors [2, 3]. Patients with tumors benefit from these local ablation therapies due to their enhanced safety, minimal invasiveness, and effectiveness [4, 5]. However, 60%–80% of tumor recurrences are observed within the 5‐year follow‐up period after local ablation treatment [6, 7]. Residual tumor cells in the periablational margin resulting from insufficient ablation are mainly responsible for tumor progression [8]. Significant efforts have been devoted to eliminating the residual tumor cells after ablation therapies [9]. Among these strategies, leveraging immunomodulators—such as cytosine‐phosphate‐guanine (CpG), anti‐lymphocyte‐activation gene 3 (LAG‐3) antibodies, and anti‐programmed cell death protein 1 (PD‐1) antibodies—to induce immune‐mediated tumor regression and eradication has emerged as a highly promising approach [10, 11, 12]. For instance, the combination of lenvatinib and sintilimab has been utilized in treating hepatocellular carcinoma following radiofrequency ablation, yet it demonstrated an overall response rate of approximately 40% and a complete response rate ranging from 3% to 13% [13]. New adjuvant immunotherapy strategies inspired by previously undisclosed mechanisms are urgently warranted to enhance the long‐term outcomes of ablation therapies.

The majority of the advanced immunotherapies currently in development primarily rely on modulating the tumor immune microenvironment to elicit an effective anti‐tumor response [14, 15, 16]. The lymphatic system [17], particularly the tumor‐draining lymph nodes (TDLNs) [18], plays a pivotal role in antigen presentation and immune activation [19, 20], thus holding strategic significance in tumor immunotherapy [21, 22, 23]. However, the alterations in the lymphatic microenvironment following insufficient tumor ablation remain largely unexplored. Loco‐regional ablation treatment methods typically preserve the integrity of lymph nodes. Merely modulating the tumor immune microenvironment while disregarding the lymphatic immune microenvironment may limit the efficacy of postablative antitumor immunotherapy.

Here, we characterize the immune landscape of TDLNs following incomplete MWA (iMWA) utilizing an experimental breast cancer model and single‐cell RNA sequencing (scRNA‐seq) analysis. Our results revealed that iMWA induces a marked expansion of multiple heterogeneous macrophage subpopulations within TDLNs, accompanied by transcriptional features associated with immune suppression, thereby contributing to the establishment of an immunosuppressive lymphatic niche. We have previously reported that iMWA facilitates the recruitment of M2‐like macrophages into residual tumors, which in turn promotes tumor recurrence [24, 25]. Based on these observations, we hypothesized that macrophages enriched in both TDLNs and residual tumors after iMWA can orchestrate tumor progression through multiple complementary mechanisms. Furthermore, the expression of colony‐stimulating factor 1 receptor (*Csf1r*) is elevated in the immunosuppressive, heterogeneous macrophage subpopulations within TDLNs after iMWA. As the CSF1‐CSF1R signaling axis is a central regulator of macrophage biology, governing their recruitment, survival, and immunoregulatory polarization [26], targeting this axis has the potential to modulate macrophage‐driven immunosuppression in both TDLNs and tumors, thereby enhancing post‐ablation antitumor immunity.

With the rapid development of nanotechnology, functionalized nanomaterials can be effectively utilized to address the limited accumulation of intravenously administered immunomodulators at targeted sites. Albumin, a multifunctional protein renowned for its exceptional drug‐binding capacity and biosafety, can leverage its intrinsic lymph node‐reflux mechanism to deliver liposomal drug formulations to the lymph nodes [27]. Leveraging this property, we developed an albumin‐based lipoprotein nanosystem by fusing albumin with polyethylene glycol (PEG)‐modified liposomes, enabling efficient delivery of therapeutic agents to TDLNs and to tumors via the enhanced permeability and retention effect. By co‐delivering paclitaxel (PTX) and the CSF1R inhibitor BLZ945, this nanosystem (PTX/BLZ945@Alb‐lip) promotes repolarization of M2 macrophages toward the M1 phenotype, thereby effectively remodeling the immune microenvironment in both lymph nodes and tumors (Figure 1). This innovative approach leads to regression or eradication of primary tumors following iMWA and also confers robust long‐term immunological memory protection in treated mice.

**FIGURE 1:**
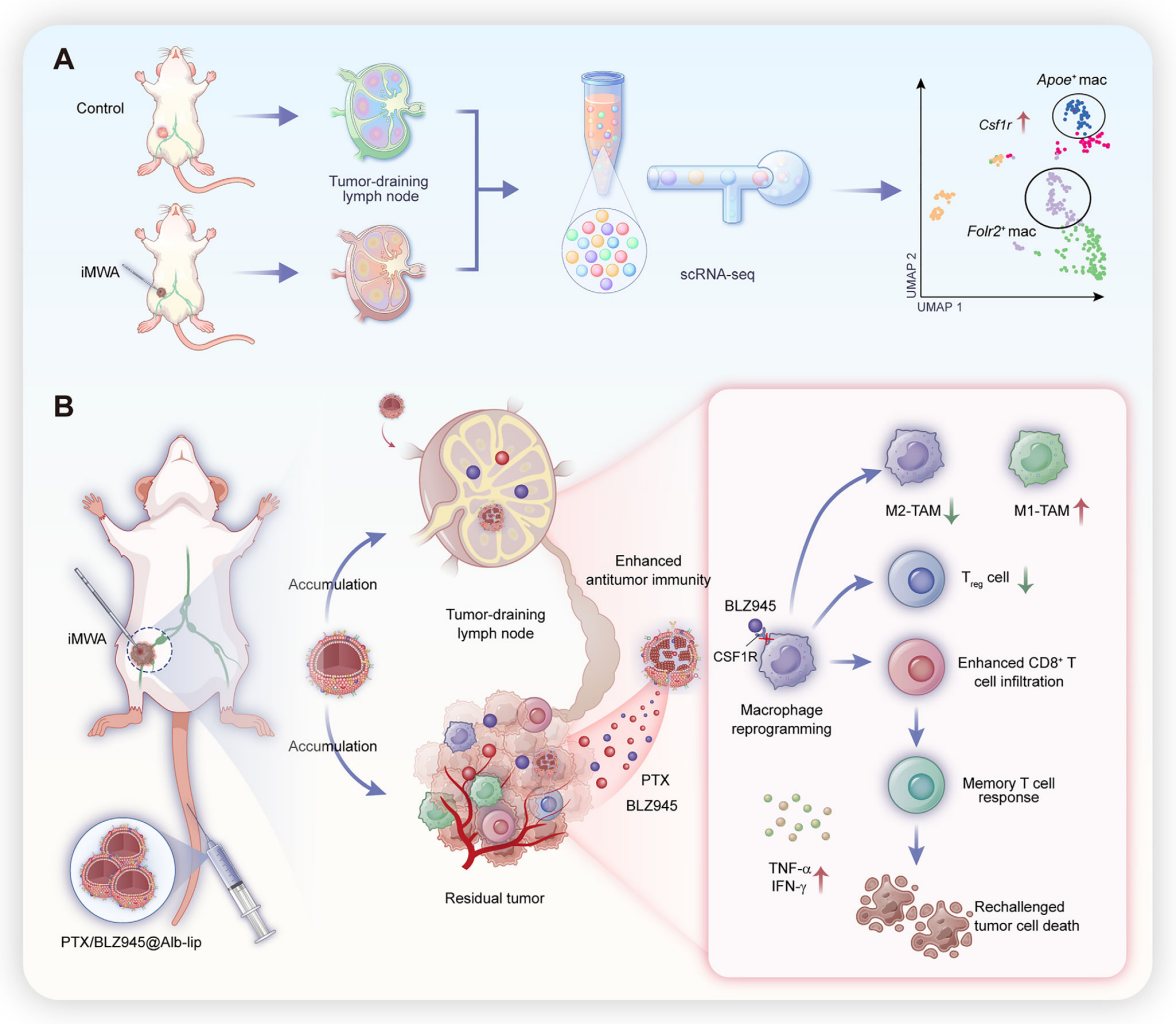
Design and mechanism of the albumin‐based lipoprotein nanosystem for enhancing post‐ablation antitumor immunity. (A) Target identification was achieved through single‐cell RNA sequencing (scRNA‐seq) analysis of tumor‐draining lymph nodes (TDLNs) following incomplete microwave ablation (iMWA), which revealed the expansion of immunosuppressive heterogeneous macrophage subpopulations with elevated *Csf1r* expression. (B) Following intravenous administration, the PTX/BLZ945@Alb‐lip nanosystem accumulates in both TDLNs and tumors and modulates macrophage activation states to reprogram the immune microenvironment. This leads to the regression or eradication of primary tumors following iMWA and also confers robust long‐term immunological memory protection in treated mice. M1‐TAM, M1‐like tumor‐associated macrophage; M2‐TAM, M2‐like tumor‐associated macrophage; Treg, regulatory T cell.

Notably, studies on TDLN resection highlight the critical importance of restoring TDLN immune function to elicit robust antitumor immunity. The direct trafficking of immunomodulators to lymph nodes elucidates a previously unexplored pathway for enhancing the antitumor immune response after ablation therapies, and our findings have significant implications for the field of cancer immunotherapy. Given the pivotal role of locoregional ablation treatment in managing a diverse range of patients with solid malignancies, our proposed post‐ablation antitumor immunotherapy approach exhibits substantial potential to establish a novel standard‐of‐care paradigm within the field of interventional oncology.

## 2 Results

### 2.1 Specialized Macrophage Subsets Contribute to the Establishment of an Immunosuppressive Lymphatic Niche After iMWA

To investigate how iMWA reshapes the immune landscape of TDLNs, we established an orthotopic 4T1 breast tumor model in BALB/c mice. Tumors were partially ablated upon reaching an appropriate size, and TDLNs were collected three days post‐treatment for scRNA‐seq analysis (Figure 2A). After quality‐control filtering, a total of 20 665 immune cells from both the control and iMWA groups were retained for subsequent integrated analysis. Unsupervised clustering based on canonical immune markers identified multiple major immune cell populations, including T cells, B cells, macrophages, natural killer (NK) cells, plasma cells, and neutrophils (Figure 2B,C). To further examine how iMWA alters immune organization within TDLNs, we applied CellChat analysis to infer intercellular communication networks. Compared with the control group, iMWA increased the overall interaction strength among immune cell populations within TDLNs (Figure 2D). Notably, macrophages exhibited extensive predicted interactions with T cells, NK cells, and neutrophils (Figures 2E and S1), suggesting their central role in post‐ablation immune remodeling.

**FIGURE 2:**
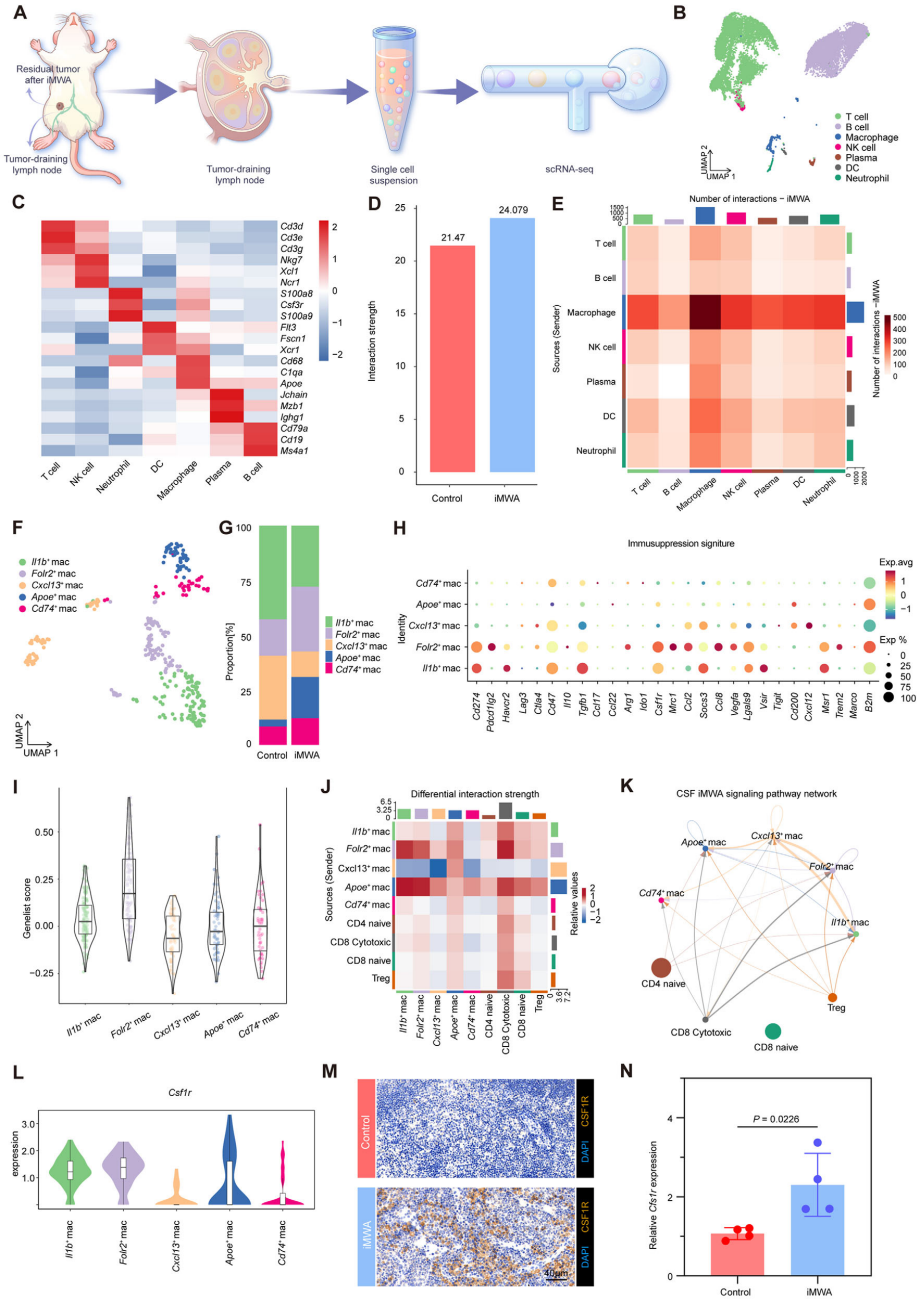
Local incomplete microwave ablation (iMWA) reshapes macrophage states in tumor‐draining lymph nodes (TDLNs). (A) Schematic illustration of the experimental workflow for single‐cell RNA sequencing (scRNA‐seq) analysis of TDLNs following iMWA. (B) Uniform Manifold Approximation and Projection (UMAP) visualization of the major immune cell populations identified in TDLNs. (C) Heatmap showing representative marker genes for each immune cell population. (D) Overall cell–cell communication strength inferred by CellChat analysis. (E) Heatmap showing the predicted cell–cell communication network among immune cell populations inferred by CellChat analysis. (F) UMAP visualization of five macrophage subsets identified in TDLNs. (G) Relative proportions of macrophage states in control and iMWA groups. (H) Bubble plot showing the expression of immunosuppressive genes across five macrophage subsets. (I) Immunosuppressive scores of five macrophage subsets. (J) Heatmap showing interaction strength between macrophage states and T‐cell populations. (K) CellChat network showing CSF signaling interactions in the iMWA group. (L) Relative expression levels of *Csf1r* across distinct macrophage states identified in TDLNs. (M) Representative immunohistochemical images showing CSF1R expression (yellow) in TDLNs (scale bar = 40 µm). (N) Quantitative polymerase chain reaction (qPCR) validation of *Csf1r* expression in TDLNs (*n* = 4). Data are presented as mean ± standard deviation (SD). Statistical significance was determined using Student's *t*‐test.

We next investigated macrophage heterogeneity in TDLNs. Sub‐clustering analysis identified five transcriptionally distinct macrophage subsets, including *Il1b^+^*, *Folr2^+^*, *Cxcl13^+^*, *Apoe^+^*, and *Cd74^+^* populations (Figures 2F and S2 and Table S1). These macrophage subsets exhibited distinct functional characteristics. For instance, *Il1b^+^* macrophages exhibited a pro‐inflammatory phenotype, characterized by the expression of *Il1b*, *Tnf*, and *S100a8*, and enrichment of inflammatory and interferon‐responsive pathways. *Folr2^+^* macrophages showed elevated expression of genes associated with tissue remodeling and immunosuppressive functions, such as *Folr2*, *Mrc1*, and *Cd163* (Figure S3). *Apoe^+^* macrophages were enriched in pathways related to negative regulation of macrophage activation and regulatory T cell differentiation, indicating a potential immunoregulatory or immunosuppressive tendency (Figure S4). Notably, several macrophage subsets, particularly *Folr2^+^* and *Apoe^+^* populations, were expanded following iMWA (Figure 2G) and were enriched in immunosuppressive transcriptional programs (Figures S3 and S4), thereby contributing to the establishment of an immunosuppressive lymphatic niche.

To systematically evaluate the immunosuppressive functional characteristics of macrophage subsets, we curated a panel of more than 30 immunosuppression‐associated genes—including *Fap, Siglece, Il10, Isg15, Arg1*, and *Ido1*—from established literature [28] and analyzed their expression across distinct macrophage subsets (Figure 2H). By applying AddModuleScore analysis to quantify the immunosuppressive signature, we found that *Folr2^+^* and *Apoe^+^* macrophages exhibited elevated immunosuppressive signatures, with *Folr2^+^* macrophages showing the highest immunosuppressive scores among all macrophage subsets (Figure 2I). Furthermore, intercellular communication analysis demonstrated extensive predicted interactions between *Folr2^+^*/*Apoe^+^* macrophages and T cell subsets (Figures 2J and S5), suggesting their potential role in modulating adaptive immune responses. Among the inferred signaling pathways, CSF signaling emerged as one of the most prominently enriched macrophage–associated communication axis. Compared with the control group, the iMWA group exhibited enhanced CSF‐associated interactions, with macrophage subsets (including *Folr2^+^* and *Apoe^+^* populations) acting as major signal recipients within the network (Figure 2K), whereas the control group showed a weaker and less connected signaling pattern (Figure S6). This pattern suggests enhanced immune cell–mediated signaling targeting macrophages within the TDLN microenvironment. Given the established role of the CSF1‐CSF1R axis in promoting macrophage survival and immunosuppressive polarization [29, 30], these findings suggest that enhanced CSF signaling may contribute to the expansion and functional activation of immunosuppressive macrophage subsets, consistent with its known role in shaping immunosuppressive immune microenvironments, thereby influencing T cell responses.

Motivated by the prominent activation of CSF signaling and the central role of macrophages as signal recipients, we next examined the expression of the key CSF receptor, *Csf1r*, across macrophage subsets. We found that *Csf1r* was broadly co‐expressed among the aforementioned specialized macrophage subsets (Figure 2L). Immunohistochemistry (IHC) and quantitative polymerase chain reaction (qPCR) analyses further verified a significant upregulation of *Csf1r* expression in TDLNs following iMWA (Figure 2M,N).

Collectively, these results demonstrate that iMWA substantially reshapes macrophage composition within TDLNs, increasing immunosuppressive macrophage subsets and upregulating *Csf1r* expression. CSF1R is a critical regulator of the monocyte–macrophage lineage and plays essential roles in macrophage survival, differentiation, and functional polarization [29]. In the tumor microenvironment, activation of the CSF1‐CSF1R signaling axis has been shown to promote the expansion and maintenance of immunosuppressive macrophages, thereby contributing to the establishment of an immunosuppressive niche [30]. These findings imply that targeting the CSF1R signaling pathway can reprogram the immune microenvironment by modulating macrophage function, thereby enhancing antitumor immunity following insufficient ablation.

To further evaluate the clinical relevance of the CSF1‐CSF1R axis identified in our mouse model, we analyzed three independent human breast cancer scRNA‐seq datasets (GSE167036, GSE225600, and GSE180286) together with The Cancer Genome Atlas (TCGA) Breast Invasive Carcinoma (BRCA) cohort. All human samples were obtained from patients without prior thermal ablation. Mouse‐derived *Folr2^+^* and *Apoe^+^* macrophage signatures were detectable in human breast cancer macrophages (Figure S7A,B). Ligand–receptor analysis further demonstrated that CSF1 was predominantly expressed by epithelial and stromal cells, whereas CSF1R was largely restricted to macrophages, both in primary tumors and lymph nodes (Figure S7C,F). In TCGA‐BRCA, CSF1R expression strongly correlated with macrophage infiltration (Figure S7D,E), and human CSF1R‐high macrophages preferentially expressed multiple immunoregulatory markers (Figure S7G). Together, these analyses support the clinical relevance of the CSF1‐CSF1R axis as a potential therapeutic target in human breast cancer.

### 2.2 CSF1R Inhibition Combined With Paclitaxel Functionally Reprograms Macrophage Activation and Inhibits Tumor Cell Growth

Given the aforementioned crucial role of the CSF1‐CSF1R signaling axis in immunosuppressive macrophage subpopulations within TDLNs, BLZ945, a CSF1R inhibitor, was introduced into this study. Previous studies have suggested that certain chemotherapeutic agents can influence macrophage activation states in addition to their direct cytotoxic effects [31]. Therefore, we carried out a screening process to identify the optimal combination of BLZ945 and chemotherapeutic agents that can effectively modulate the functional phenotype of macrophages.

Gemcitabine (GEM), doxorubicin (DOX), and PTX were selected as representative candidate chemotherapeutic agents because they are clinically established cytotoxic drugs recommended by the current National Comprehensive Cancer Network (NCCN) and European Society for Medical Oncology (ESMO) breast cancer guidelines, representing three distinct classes of chemotherapy, namely nucleoside analogs, anthracyclines, and taxanes, respectively [32, 33]. These representative agents were therefore selected to systematically evaluate their compatibility with the CSF1R inhibitor BLZ945 and identify an optimal combination capable of enhancing macrophage immunomodulation.

RAW264.7 macrophages were first stimulated with interleukin‐4 (IL‐4) and interleukin‐13 (IL‐13), and then treated with different drug combinations (Figure 3A). Among the tested conditions, the combination of BLZ945 and PTX induced the most pronounced shift in macrophage activation‐associated cytokine profiles compared to treatment with either BLZ945 or PTX alone. Specifically, the secretion of M1 markers—including TNF‐α and IL‐12—increased, whereas the expression of M2 markers such as IL‐10 and TGF‐β decreased (Figures 3B and S8). However, the combinations of BLZ945 with other chemotherapeutic agents (gemcitabine or doxorubicin) did not show such an effect.

**FIGURE 3:**
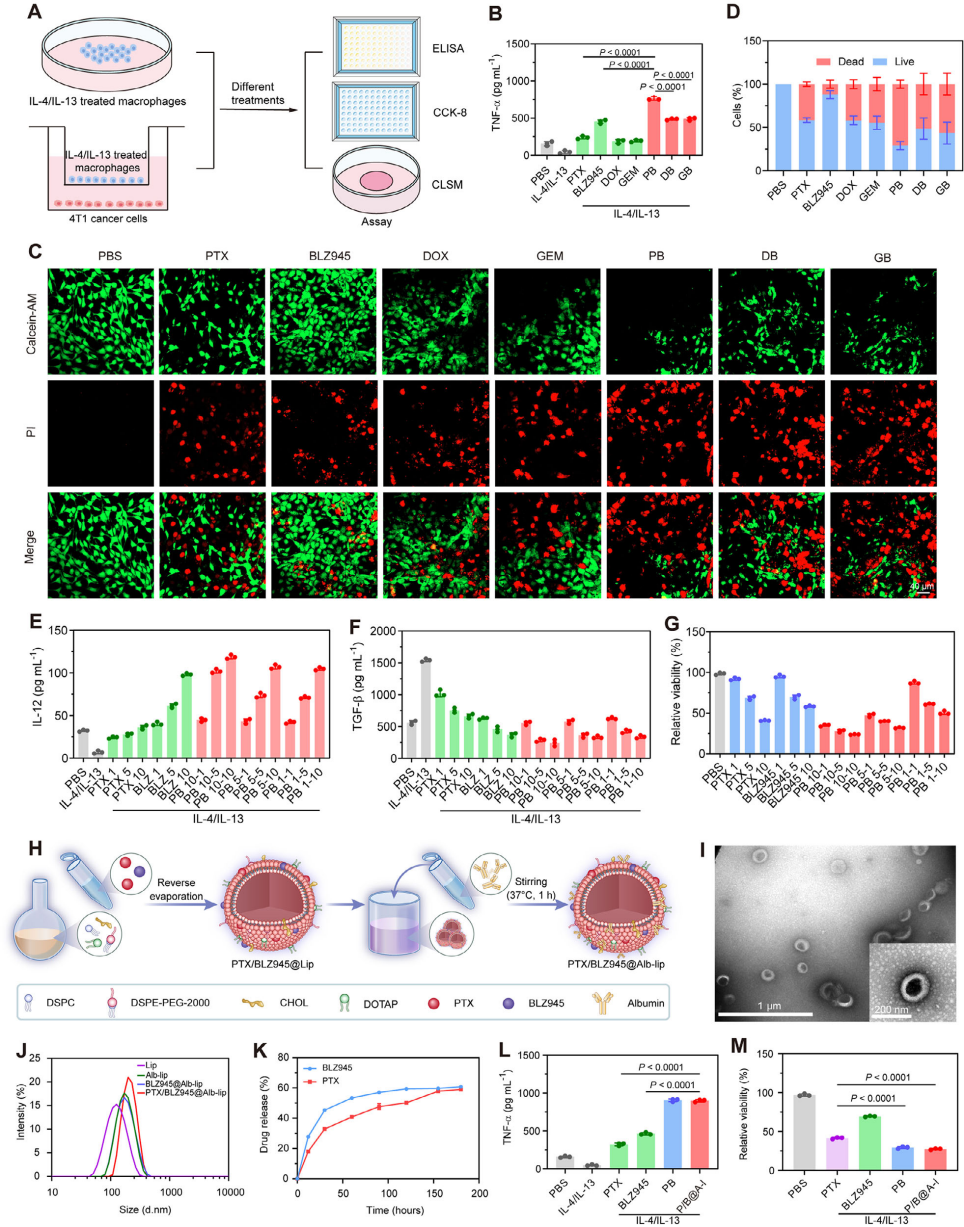
Development and characterization of PTX/BLZ945@Alb‐lip for macrophage functional reprogramming and tumor cell inhibition. (A) Schematic of the in vitro experimental design. (B) Tumor necrosis factor‐α (TNF‐α) secretion from RAW264.7 macrophages after different treatments. (C, D) Representative confocal laser scanning microscopy (CLSM) images (C) and quantification (D) of live/dead 4T1 cells co‐cultured with treated macrophages (scale bar = 40 µm). (E, F) Interleukin‐12 (IL‐12) (E) and transforming growth factor‐β (TGF‐β) (F) secretion from treated macrophages. (G) Viability of 4T1 cells co‐cultured with treated macrophages. (H) Schematic of PTX/BLZ945@Alb‐lip preparation. (I) Representative transmission electron microscopy (TEM) image of PTX/BLZ945@Alb‐lip (scale bars, 1 µm and 200 nm). (J) Dynamic light scattering (DLS) size distributions of the indicated formulations. (K) In vitro cumulative release profiles of PTX and BLZ945 from PTX/BLZ945@Alb‐lip. (L) TNF‐α secretion from macrophages following different treatments. (M) Viability of 4T1 cells co‐cultured with treated macrophages. PTX, paclitaxel; BLZ945, a colony‐stimulating factor 1 receptor (CSF1R) inhibitor; DOX, doxorubicin; GEM, gemcitabine; PB, PTX + BLZ945; DB, DOX + BLZ945; GB, GEM + BLZ945; P/B@A‐l, PTX/BLZ945@Alb‐lip. Data are presented as mean ± SD (*n* = 3). Statistical significance was determined using one‐way ANOVA followed by Tukey's multiple comparisons test.

To further evaluate whether the modulation of macrophages can influence the behavior of tumor cells, we established a co‐culture system composed of 4T1 breast cancer cells and macrophages. The combination of BLZ945 and PTX demonstrated the most potent inhibitory effect on the growth of 4T1 tumor cells, while the co‐administration of BLZ945 with gemcitabine and doxorubicin did not exhibit significant synergistic enhancement compared to individual drug treatments (Figures 3C,D and S9). Notably, the inhibitory effect was more pronounced in the macrophage co‐culture system than in tumor cells cultured alone, suggesting that macrophage‐associated mechanisms may contribute to the observed antitumor activity. Moreover, the extent of macrophage repolarization and tumor cell suppression varied depending on the PTX‐ to‐ BLZ945 ratio. The most pronounced effects on macrophage repolarization and tumor cell inhibition (Figures 3E–G and S10) were observed at a PTX‐ to‐ BLZ945 ratio of 2:1. Collectively, these results indicate that the combined CSF1R inhibition and PTX treatment reshapes macrophage functional programs and enhances macrophage‐associated antitumor activity.

### 2.3 Synthesis and Characterization of PTX/BLZ945@Alb‐lip

To effectively reprogram macrophage states, it is crucial to efficiently deliver small molecules to both lymph nodes and tumors where the macrophages are located. The aqueous solubility of free BLZ945 and PTX is extremely low, which further constrains their clinical efficacy. To address these challenges, we proposed an engineered lipoprotein nanosystem (Figure 3H). The nanosystem comprises albumin in conjunction with PEG‐modified liposomes, which not only efficiently encapsulate BLZ945 and PTX but also enhance biocompatibility and stability. Transmission electron microscopy (TEM) indicated that PTX/BLZ945@Alb‐lip exhibited a uniform spherical form (Figure 3I). Dynamic light scattering (DLS) revealed that the average hydrodynamic diameter of PTX/BLZ945@Alb‐lip was below 200 nanometers (Figure 3J).

The increase in particle size and the decrease in zeta potential observed from Lip to Alb‐lip indicate the successful loading of albumin (Figures 3J and S11). Also, the sodium dodecyl sulfate‐polyacrylamide gel electrophoresis (SDS‐PAGE) analysis revealed that the protein bands of PTX/BLZ945@Alb‐lip exhibited an identical distribution pattern to pure albumin, indicating successful loading of albumin (Figure S12). In the ultraviolet–visible (UV–vis) absorption spectrum of PTX/BLZ945@Alb‐lip, a prominent absorption peak of BLZ945 at ∼273 nm was detected, signifying that BLZ945 has been successfully encapsulated within PTX/BLZ945@Alb‐lip (Figure S13). The successful co‐loading of PTX was confirmed by high‐performance liquid chromatography (HPLC) (Figure S14).

Upon incubation in phosphate‐buffered saline (PBS) containing 10% serum at 4°C for 7 days, the size of PTX/BLZ945@Alb‐lip showed no significant variation, and PTX/BLZ945@Alb‐lip sustained a low polydispersity index (PDI), which indicates that PTX/BLZ945@Alb‐lip exhibits excellent stability (Figure S15). The loading efficiencies and capacities of PTX and BLZ945 within PTX/BLZ945@Alb‐lip fluctuated in accordance with alterations in the initial feed concentration (Figure S16). The nanosystem employed in this study achieves sustained release of the therapeutics, and after 180 h, the cumulative release rate of BLZ945 and PTX within the engineered lipoprotein nanosystem reached 60% (Figure 3K).

Benefiting from the favorable physical properties, high stability, and advantageous drug release characteristics, the lipoprotein nanosystem employed in this study is expected to optimize BLZ945/PTX utilization and enhance antitumor efficacy. We next tested the PTX/BLZ945@Alb‐lip nanosystem in vitro. As expected, the treatment of PTX/BLZ945@Alb‐lip augmented the functional reprogramming of immunoregulatory macrophages toward a more pro‐inflammatory activation state, as demonstrated by the results shown in Figures 3L and S17. Additionally, it effectively inhibited the growth of 4T1 breast cancer cells (Figure S18). Moreover, in comparison to 4T1 breast cancer cells cultured independently, the inhibitory effect of PTX/BLZ945@Alb‐lip on 4T1 breast cancer cells co‐cultured with macrophages was significantly enhanced (Figure 3M). This suggests that the antitumor activity of PTX/BLZ945@Alb‐lip is reliant on macrophages. To exclude the possibility that the observed immunomodulatory effects resulted from nonspecific immune cell toxicity, we further evaluated the cytocompatibility of PTX/BLZ945@Alb‐lip using RAW264.7 macrophages and mouse bone marrow‐derived dendritic cells (BMDCs). At the therapeutic concentrations used in this study, neither free drugs nor PTX/BLZ945@Alb‐lip caused significant cytotoxicity, and cell viability remained above 85% in both cell types (Figure S19), indicating good biocompatibility toward immune cells.

### 2.4 PTX/BLZ945@Alb‐lip Enhances Drug Accumulation in Both TDLNs and Tumors

Consequently, we evaluated the potential of intravenous administration of PTX/BLZ945@Alb‐lip to enhance drug accumulation in both TDLNs and tumors compared to conventional liposomes. The in vivo imaging system (IVIS) was employed to perform region of interest (ROI) quantitative analysis, aiming to monitor the accumulation of Cy5.5‐labeled PTX/BLZ945@Alb‐lip in both lymph nodes and tumors of tumor‐bearing mice. As expected, in contrast to PTX/BLZ945@Lip, PTX/BLZ945@Alb‐lip exhibited a more pronounced accumulation in lymph nodes and tumor sites following intravenous administration via the tail vein, with the peak fluorescence intensity observed at 8 h post‐administration (Figure 4A,B). Notably, the PTX/BLZ945@Alb‐lip formulation exhibited prolonged retention in lymph nodes and tumor sites, with a duration of up to 48 h.

**FIGURE 4:**
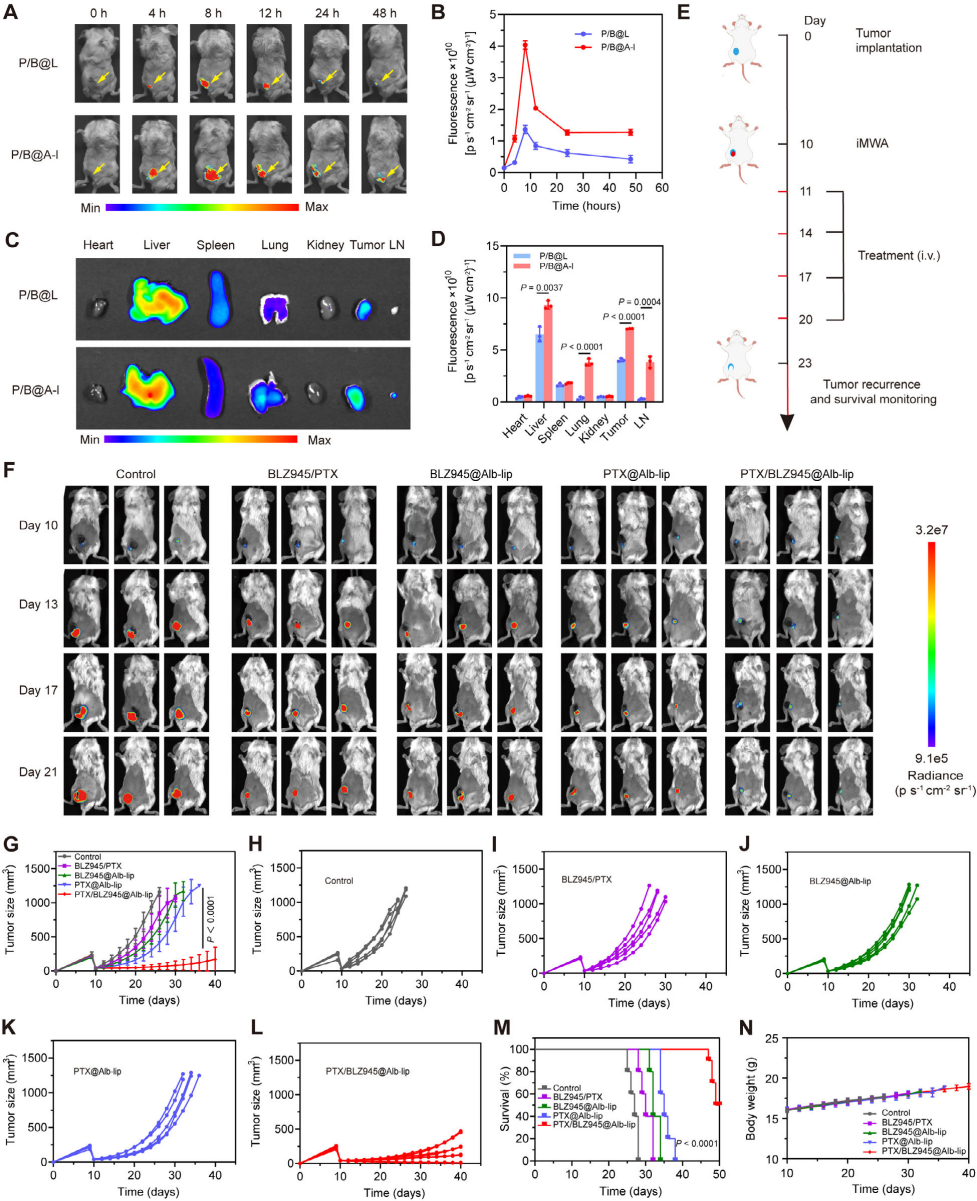
PTX/BLZ945@Alb‐lip enhances drug accumulation in tumor‐draining lymph nodes (TDLNs) and tumors and inhibits post‐ablation tumor progression. (A, B) In vivo fluorescence imaging (A) and quantitative fluorescence analysis (B) following intravenous administration of Cy5.5‐labeled PTX/BLZ945@Lip or PTX/BLZ945@Alb‐lip. Yellow arrows indicate the locations of lymph nodes and tumors. (C, D) Ex vivo fluorescence imaging (C) and quantitative analysis (D) of major organs, tumors, and TDLNs collected 8 h after administration. (E) Schematic illustration of the in vivo therapeutic protocol. (F) Representative bioluminescence images showing tumor progression following different treatments. (G–L) Mean tumor growth curves (G) and individual tumor growth curves (H–L) in mice receiving different treatments. (M) Kaplan–Meier survival analysis. (N) Body weight changes during treatment. P/B@L, PTX/BLZ945@Lip; P/B@A‐l, PTX/BLZ945@Alb‐lip; LN, lymph node. Data are presented as mean ± SD. *n* = 3 mice per group in (A–D); *n* = 10 mice for the PTX/BLZ945@Alb‐lip group and *n* = 5 mice for all other groups in (F–N). Statistical significance was determined using one‐way ANOVA followed by Tukey's multiple comparisons test. Survival curves were analyzed using the log‐rank (Mantel–Cox) test.

The TDLNs, tumors, and major organs (including the heart, liver, spleen, lung, and kidney) were collected from the mice 8 h after injection for subsequent ex vivo fluorescence imaging and ROI quantification. As shown in Figure 4C,D, fluorescence signals from PTX/BLZ945@Alb‐lip were notably stronger in both TDLNs and tumors compared with conventional liposomes, while relatively lower signals were observed in most normal organs. These findings indicate that the albumin‐based nanosystem facilitates efficient delivery and retention of therapeutic agents within both lymphatic tissues and tumor sites.

To further quantitatively validate the biodistribution of the nanosystem, PTX and BLZ945 concentrations in tumors and TDLNs were determined by liquid chromatography–tandem mass spectrometry (LC‐MS/MS) 8 h after intravenous administration. Consistent with the fluorescence imaging results, PTX/BLZ945@Alb‐lip markedly increased the accumulation of both PTX and BLZ945 in tumors and TDLNs compared with PTX/BLZ945@Lip (Figure S20), providing quantitative evidence that albumin‐coated liposomes enhance drug accumulation in both tumors and TDLNs following intravenous administration.

To further histologically evaluate lymphatic‐associated delivery, TDLNs were collected 8 h after intravenous administration of Cy5‐labeled liposomes for confocal imaging. Compared with uncoated liposomes, PTX/BLZ945@Alb‐lip exhibited stronger Cy5 fluorescence and preferential localization within LYVE‐1‐positive lymphatic sinus regions of TDLNs (Figure S21), providing histological evidence supporting enhanced lymphatic‐associated delivery of albumin‐coated liposomes following intravenous administration.

### 2.5 PTX/BLZ945@Alb‐lip Inhibits Postablative Tumor Progression

Next, the therapeutic efficacy of PTX/BLZ945@Alb‐lip in preventing post‐ablation tumor progression was evaluated. Ten days after inoculation with 4T1 cancer cells, partial tumor ablation was performed using iMWA. Then, the tumor‐bearing mice were randomly divided into five groups before treatment, including PBS, BLZ945/PTX, BLZ945@Alb‐lip, PTX@Alb‐lip, and PTX/BLZ945@Alb‐lip. The mice were intravenously injected with PBS, BLZ945/PTX, BLZ945@Alb‐lip, PTX@Alb‐lip, or PTX/BLZ945@Alb‐lip according to the groups (dosages of BLZ945 at 5 mg kg^−1^ and PTX at 10 mg kg^−1^ per mouse) (Figure 4E). Tumor growth was monitored by capturing bioluminescence signals emitted from 4T1 tumor cells (Figure 4F). Our findings revealed that the administration of free BLZ945/PTX at the experimental dosage did not exhibit a significant suppressive effect on tumor growth. Although PTX@Alb‐lip alone induced a partial delay in tumor growth, the residual tumors treated with PTX/BLZ945@Alb‐lip exhibited the slowest rate of outgrowth and all mice survived throughout the 40‐day observation period, with complete responses observed in 40% of mice (Figure 4G–M). The body weight of mice in the PTX/BLZ945@Alb‐lip treatment group did not show any abnormal alterations compared to the other treatment groups (Figure 4N).

### 2.6 Mechanisms Underlying the Antitumor Responses Induced by PTX/BLZ945@Alb‐lip

To further clarify the mechanisms underlying the antitumor responses induced by PTX/BLZ945@Alb‐lip in the post‐ablation setting, all mice included in the experiments shown in Figure 5 first underwent iMWA and were subsequently treated with PBS, free BLZ945/PTX, BLZ945@Alb‐lip, PTX@Alb‐lip, or PTX/BLZ945@Alb‐lip, as illustrated in the experimental timeline (Figure 4E). TDLNs and residual tumors were collected on day 23 after tumor implantation for flow cytometric, multiplex immunofluorescence (mIF), and immunohistochemical analyses. In contrast to other groups, we observed variations in diverse immune cell populations within the lymph nodes and tumors of mice treated with PTX/BLZ945@Alb‐lip. Specifically, when compared with other treatment groups, the PTX/BLZ945@Alb‐lip group manifested a significant reduction in M2‐TAMs, an increase in M1‐TAMs, an elevation in CD8^+^ T cells, and a decrease in regulatory T cells (Tregs) within the TDLNs (Figures 5A–F and S22) and tumors (Figures S23–S26). Particularly, the M1/M2 and CD8^+^ T cell/Treg ratios, which are regarded as indicators of antitumor immune homeostasis, were significantly enhanced in the lymph nodes (Figure 5G,H) and tumors (Figures S27 and S28) of the PTX/BLZ945@Alb‐lip group. Additionally, the levels of interferon‐γ (IFN‐γ) and TNF‐α were elevated in the serum of tumor‐bearing mice treated with PTX/BLZ945@Alb‐lip (Figure 5I,J), further confirming that this strategy elicits a potent immune response. The flow cytometry results were further corroborated by mIF analysis (Figure 5K). Ki‐67 expression in tumors was significantly reduced in the PTX/BLZ945@Alb‐lip group (Figure S29), indicating that the engineered lipoprotein drug delivery system effectively suppresses tumor proliferation. In addition, compared with other groups, the expression of CSF1R in the lymph nodes and tumors of the PTX/BLZ945@Alb‐lip group was significantly downregulated (Figures 5L and S30). To provide additional anatomical context for the immunostaining analyses, low‐magnification whole‐section images of the corresponding TDLN specimens were additionally examined. The overview images demonstrated the intact tissue architecture and indicated the locations of the representative high‐magnification regions shown in Figures 5K,L and S31.

**FIGURE 5:**
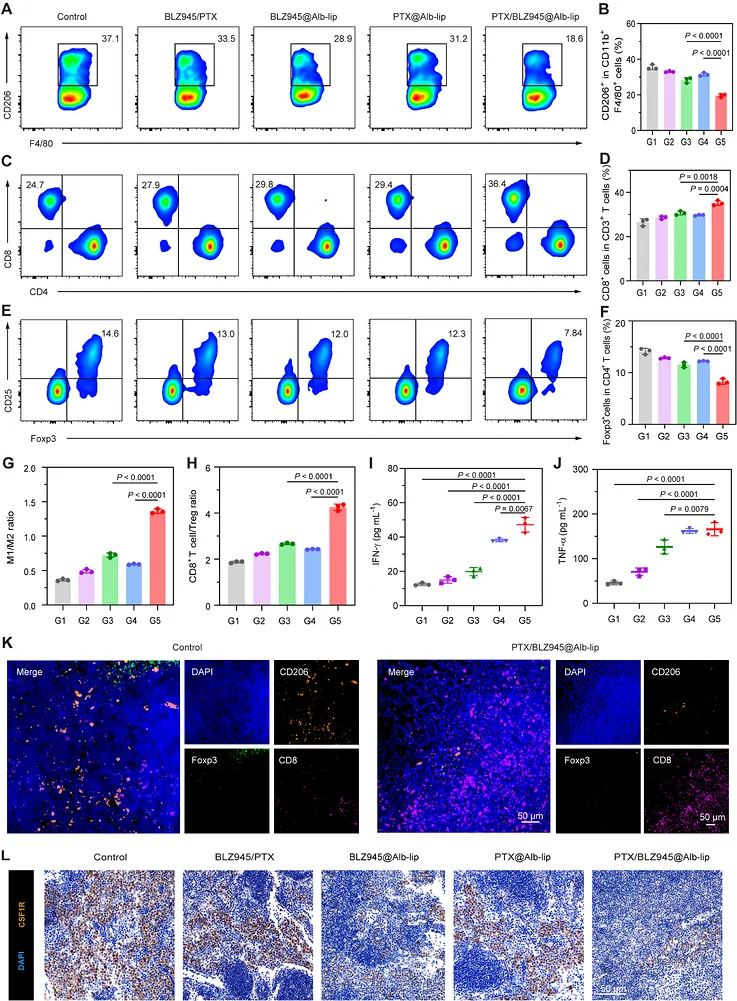
Mechanisms underlying the antitumor responses induced by PTX/BLZ945@Alb‐lip following iMWA. (A, B) Representative flow cytometry (A) and quantitative analysis (B) of M2‐TAMs (CD206^+^CD11b^+^F4/80^+^CD45^+^) within lymph nodes. (C, D) Representative flow cytometry (C) and quantitative analysis (D) of CD8^+^ T cells (CD8^+^CD3^+^CD45^+^) within lymph nodes. (E, F) Representative flow cytometry (E) and quantitative analysis (F) of Tregs (Foxp3^+^CD25^+^CD4^+^CD3^+^CD45^+^) within lymph nodes. Relevant gating schemes were shown in Figures S38 and S39. (G) Quantification of the M1/M2 ratio in lymph nodes by flow cytometry. (H) Quantification of the CD8^+^ T cell/Treg ratio in lymph nodes by flow cytometry. (I, J) The levels of IFN‐γ (I) and TNF‐α (J) in the serum of tumor‐bearing mice after various treatments. (K) Representative high‐magnification multiplex immunofluorescence images of TDLNs (scale bar = 50 µm). (L) Representative high‐magnification immunohistochemical images of CSF1R staining (yellow) in TDLNs (scale bar = 50 µm). G1, Control; G2, BLZ945/PTX; G3, BLZ945@Alb‐lip; G4, PTX@Alb‐lip; G5, PTX/BLZ945@Alb‐lip. Data are presented as mean ± SD (*n* = 3). Statistical significance was determined using one‐way ANOVA followed by Tukey's multiple comparisons test.

To further investigate whether iMWA enhances the therapeutic efficacy of PTX/BLZ945@Alb‐lip in the post‐ablation setting, we performed an additional experiment including four groups: PBS, PTX/BLZ945@Alb‐lip, iMWA + PBS, and iMWA + PTX/BLZ945@Alb‐lip. In this experiment, the first two groups received PBS or PTX/BLZ945@Alb‐lip treatment without prior iMWA, whereas the latter two groups underwent iMWA followed by PBS or PTX/BLZ945@Alb‐lip administration. PTX/BLZ945@Alb‐lip significantly inhibited tumor growth in the absence of iMWA, demonstrating its intrinsic antitumor activity. However, treatment with PTX/BLZ945@Alb‐lip following iMWA achieved the strongest suppression of tumor growth and the lowest terminal tumor weight among all groups (Figure S32A–D). Immune profiling further showed that iMWA alone induced immune alterations characterized by increased M2‐like TAMs and Tregs and decreased M1‐like TAMs and CD8^+^ T cells. Administration of PTX/BLZ945@Alb‐lip after iMWA markedly reversed these alterations, resulting in increased M1/M2 and CD8^+^ T cell/Treg ratios and elevated serum IFN‐γ and TNF‐α levels (Figures S32E–L and S33). Collectively, these findings demonstrate that PTX/BLZ945@Alb‐lip possesses intrinsic antitumor activity, while iMWA‐associated immune alterations further potentiate its immunomodulatory efficacy and therapeutic benefit in the post‐ablation setting.

To assess the potential toxic effects of the engineered lipoprotein drug delivery system, serum biochemical assays and histological analyses of major organs were performed on the third day subsequent to the last treatment among the different treatment groups. The indicators from the PTX/BLZ945@Alb‐lip group remained within the normal range, indicating that PTX/BLZ945@Alb‐lip does not elicit significant systemic toxic effects at the experimental dosage (Figures S34 and S35). The findings suggest that treatment with PTX/BLZ945@Alb‐lip effectively and safely modulates the immune microenvironment of lymph nodes and tumors, thereby enhancing postablative antitumor immunotherapy.

### 2.7 PTX/BLZ945@Alb‐lip Induces Persistent Immune Memory

Immunological memory represents a hallmark of effective cancer immunotherapy. To determine whether PTX/BLZ945@Alb‐lip treatment could induce durable immune memory responses, mice that achieved complete tumor regression after PTX/BLZ945@Alb‐lip therapy were rechallenged with 4T1 tumor cells on the contralateral side on day 50 (Figure 6A). Age‐ and sex‐matched naive mice inoculated with the same number of tumor cells served as controls. We found that the tumors in naive mice grew rapidly, whereas tumor growth in previously cured mice was markedly suppressed (Figure 6B–E). Among the four cured mice subjected to tumor rechallenge, only one developed a measurable recurrent tumor, whereas the remaining three completely rejected tumor rechallenge and remained tumor‐free throughout the observation period. Consequently, the individual tumor growth curves of these three mice completely overlapped at the baseline (tumor volume = 0 mm^3^) in Figure 6D. Notably, all mice previously treated with PTX/BLZ945@Alb‐lip survived for more than 90 days (Figure 6F), and approximately 75% (3/4) of mice exhibited complete resistance to tumor rechallenge. These results indicate that PTX/BLZ945@Alb‐lip treatment induces durable antitumor immune memory capable of preventing tumor recurrence.

**FIGURE 6:**
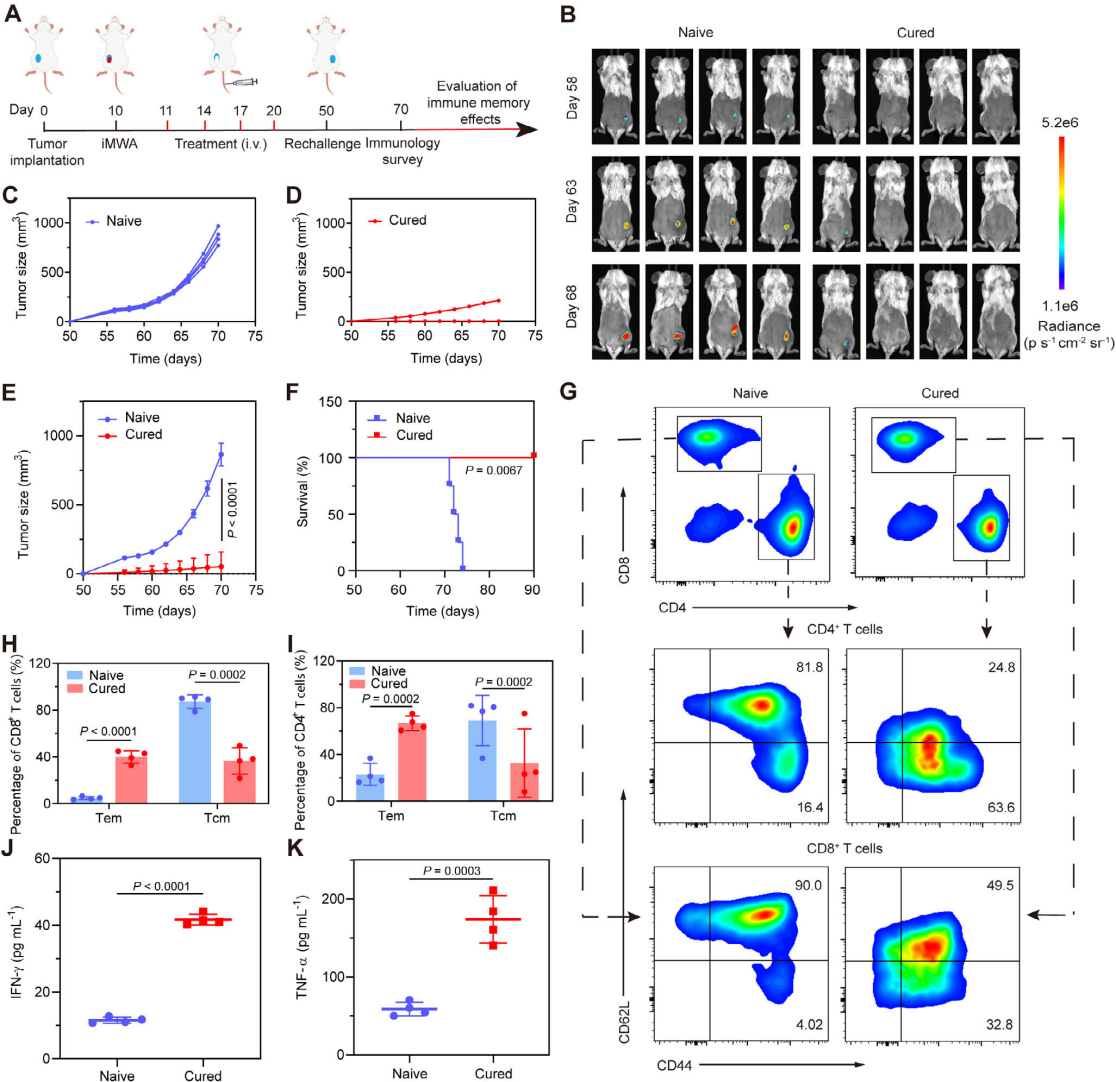
PTX/BLZ945@Alb‐lip induces persistent immune memory. (A) Schematic illustration of the experimental design for evaluating the immune memory response induced by PTX/BLZ945@Alb‐lip. (B) In vivo bioluminescence images depicting tumor progression in naive mice and cured mice. (C, D) Individual tumor growth curves of naive mice (C) and cured mice (D) following tumor rechallenge. In (D), three tumor‐free mice had overlapping curves at 0 mm^3^ and are not individually distinguishable. (E) Alterations in the mean tumor volume of naive and cured mice. (F) Survival curves of the treated mice. (G–I) Representative flow cytometry (G) and quantitative analysis (H, I) of central memory T cells (Tcm, CD62L^+^CD44^+^) and effector memory T cells (Tem, CD62L^−^CD44^+^) within CD8^+^ and CD4^+^ T‐cell populations in the spleen. Relevant gating scheme is shown in Figure S40. (J, K) Levels of IFN‐γ (J) and TNF‐α (K) in the serum of mice from naive and cured groups. Data are presented as mean ± SD (*n* = 4). Statistical significance was determined using Student's *t*‐test. Survival curves were analyzed using the log‐rank (Mantel–Cox) test.

To further characterize the immune mechanisms underlying this long‐term protection, spleen and serum samples were collected on day 20 after tumor rechallenge. Memory T‐cell populations were analyzed by flow cytometry. PTX/BLZ945@Alb‐lip–treated mice exhibited a significant expansion of effector memory T cells (Tem) within both CD8^+^ and CD4^+^ T‐cell compartments compared with naive mice, whereas central memory T cells (Tcm) showed a relatively modest change (Figure 6G–I). Consistent with enhanced immune activation, serum levels of IFN‐γ and TNF‐α were significantly elevated in PTX/BLZ945@Alb‐lip–treated mice (Figure 6J,K). Together, these findings demonstrate that PTX/BLZ945@Alb‐lip treatment induces robust and persistent antitumor immune memory responses.

### 2.8 The Critical Role of Restoring TDLN Immune Function in Enhancing Antitumor Immunity

To determine whether the therapeutic efficacy of PTX/BLZ945@Alb‐lip following iMWA depends on intact TDLNs, we performed surgical resection of TDLNs and evaluated antitumor responses (Figure 7A). Ten days after 4T1 tumor implantation, iMWA was performed and mice were randomized into four groups: control (G1), TDLN resection (G2), TDLN resection combined with PTX/BLZ945@Alb‐lip treatment (G3), and PTX/BLZ945@Alb‐lip alone (G4). Longitudinal monitoring revealed that TDLN resection alone did not suppress tumor growth. Although PTX/BLZ945@Alb‐lip treatment partially delayed tumor progression after lymph node removal, the most pronounced antitumor effect was observed in mice that received PTX/BLZ945@Alb‐lip while retaining intact TDLNs (Figure 7B–E).

**FIGURE 7:**
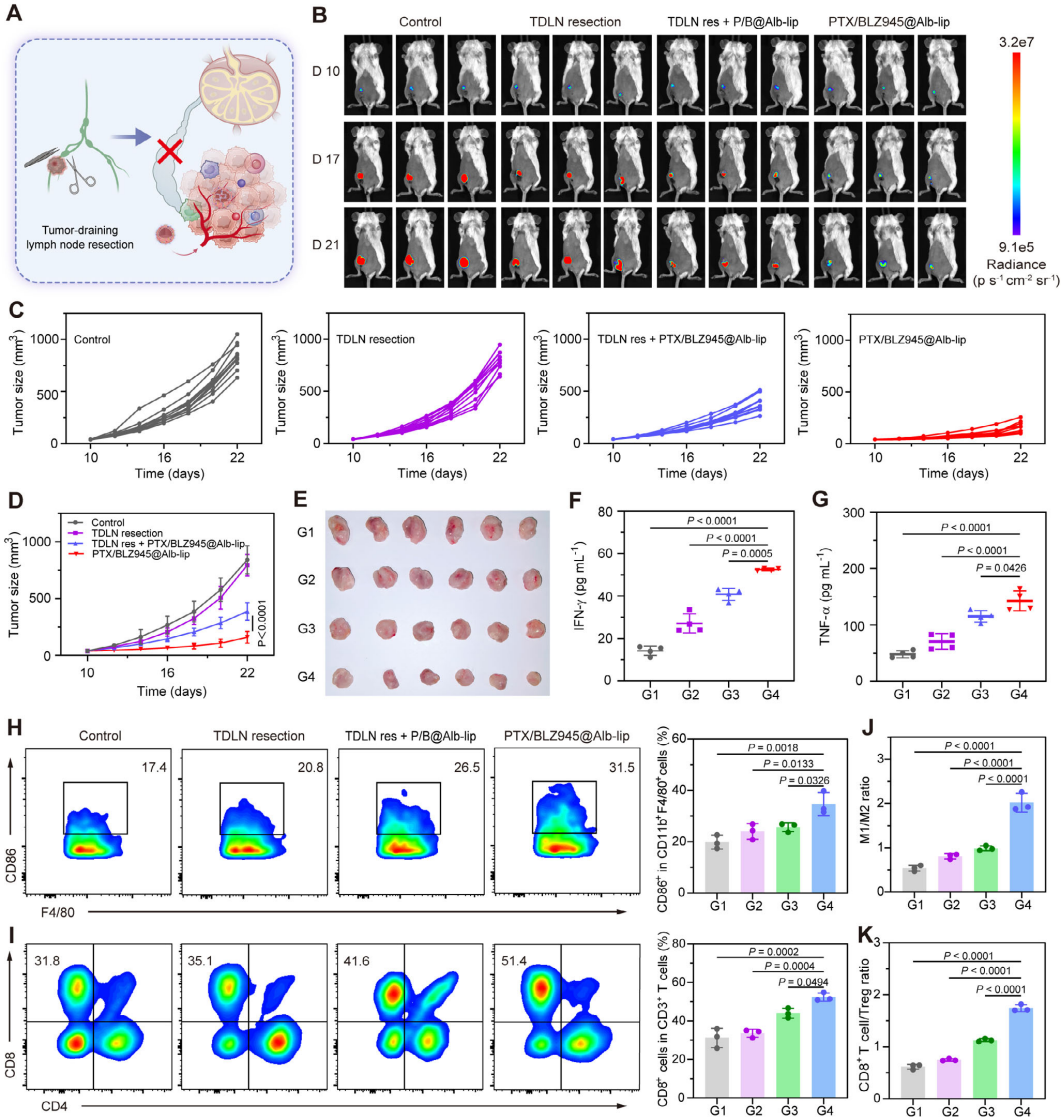
The role of TDLNs in enhancing PTX/BLZ945@Alb‐lip–mediated antitumor immunity. (A) Schematic illustration of the experimental design evaluating antitumor efficacy following TDLN resection. (B) Representative bioluminescence images monitoring tumor growth in different treatment groups. (C, D) Individual (C) and mean (D) tumor volume changes in mice following various treatments (*n* = 10). (E) Gross images of tumors in control, TDLN resection, TDLN resection + PTX/BLZ945@Alb‐lip, and PTX/BLZ945@Alb‐lip groups. (F, G) Serum levels of IFN‐γ (F) and TNF‐α (G) in tumor‐bearing mice after various treatments (*n* = 4). (H) Representative flow cytometry and quantitative analysis of M1‐TAMs (CD86^+^CD11b^+^F4/80^+^CD45^+^) within tumors (*n* = 3). (I) Representative flow cytometry and quantitative analysis of CD8^+^ T cells (CD8^+^CD3^+^CD45^+^) within tumors (*n* = 3). Relevant gating schemes are shown in Figures S38 and S39. (J) Quantification of the M1/M2 ratio in tumors by flow cytometry (*n* = 3). (K) Quantification of the CD8^+^ T cell/Treg ratio in tumors by flow cytometry (*n* = 3). G1, Control; G2, TDLN resection; G3, TDLN resection + PTX/BLZ945@Alb‐lip; G4, PTX/BLZ945@Alb‐lip. Data are presented as mean ± SD. Statistical significance was determined using one‐way ANOVA followed by Tukey's multiple comparisons test.

Mechanistically, systemic cytokine analysis revealed significantly higher levels of IFN‐γ and TNF‐α in mice treated with PTX/BLZ945@Alb‐lip with preserved TDLNs than in those subjected to TDLN resection (Figure 7F,G), indicating enhanced systemic immune activation. Flow cytometric profiling further demonstrated substantial immune remodeling within the tumors with TDLN preservation. Specifically, PTX/BLZ945@Alb‐lip treatment increased the abundance of immunostimulatory M1‐TAMs and CD8^+^ T cells, while reducing M2‐TAMs and Tregs (Figure 7H,I; Figures S36 and S37). Correspondingly, both the M1/M2 macrophage ratio and the CD8^+^ T cell/Treg ratio were significantly elevated in the TDLN‐preserved group (Figure 7J,K). Collectively, these findings demonstrate that preserving TDLNs is essential for maximizing the immunotherapeutic efficacy of PTX/BLZ945@Alb‐lip following ablation therapy. Rather than eliminating lymph nodes, therapeutic reprogramming of their immune microenvironment may represent a more effective strategy to enhance systemic antitumor immunity.

## 3 Conclusion

Currently, ablation therapies are widely endorsed in multiple clinical practice guidelines as the standard‐of‐care treatment for various tumor types due to their demonstrated advantages of exceptional safety, satisfactory efficacy, and prompt wound recovery [2, 3]. Thus, optimizing the benefits of these loco‐regional approaches remains an active area of worldwide clinical investigation. The field of systemic therapies following standard locoregional ablation treatments is rapidly advancing, with particular interest in trials incorporating immunotherapy [34]. However, mature outcomes of these combinations revealed that the objective response rates ranged only from 33% to 45% [13]. Herein, using a preclinical 4T1 breast cancer murine model, we identified an immunosuppressive landscape in TDLNs, characterized by enrichment of immune‐suppressive macrophage subsets following iMWA. Considering the crucial role of communication between tumors and lymph nodes in immunotherapy, we hypothesize that simultaneous modulation of the suppressive microenvironment in lymph nodes and tumors after ablation could enhance the efficacy of antitumor response. Our study further presents a lipoprotein nanosystem capable of delivering both PTX and BLZ945 to tumors and TDLNs, leading to comprehensive remodeling of the immune microenvironment, eradication of primary tumors following iMWA treatment, and prevention of recurrence.

Based on these findings, PTX was selected as the chemotherapeutic partner for BLZ945 for subsequent mechanistic studies and nanoplatform construction. The superior efficacy of the PTX–BLZ945 combination compared with GEM–BLZ945 and DOX–BLZ945 may be attributed to the complementary macrophage‐modulating properties of PTX and BLZ945. Beyond its cytotoxic activity, PTX has been reported to promote macrophage reprogramming toward a pro‐inflammatory M1‐like phenotype through TLR4‐dependent signaling, accompanied by enhanced TNF‐a and IL‐12 production [35, 36]. In contrast, BLZ945 suppresses CSF1R signaling and attenuates the maintenance and immunoregulatory function of CSF1R‐associated macrophages [37]. These complementary activities are likely to cooperatively remodel macrophage function by simultaneously promoting pro‐inflammatory macrophage activation while relieving CSF1R‐dependent immunosuppressive signaling. Such coordinated regulation provides a plausible mechanistic explanation for the superior cytokine profile and therapeutic efficacy of the PTX–BLZ945 combination observed in this study.

The enhanced therapeutic efficacy of PTX/BLZ945@Alb‐lip can also be attributed to the ability of the albumin‐based nanosystem to efficiently deliver both agents to tumors and TDLNs. In contrast, intravenous administration of PTX and BLZ945 as free drugs exhibited limited drug accumulation within the lymph nodes and tumors, resulting in significantly reduced anticancer efficacy. This enhanced biodistribution enabled effective remodeling of the immunosuppressive microenvironment into an immunogenic tumoricidal state within both tumors and TDLNs.

Previous studies have predominantly focused on lymph node targeting through afferent lymphatic drainage following intradermal or subcutaneous administration [38]. In contrast, our study demonstrates that intravenous administration of an albumin‐engineered lipoprotein nanosystem significantly enhances drug delivery to both residual tumors and TDLNs. The enhanced accumulation of PTX/BLZ945@Alb‐lip in TDLNs may be attributed to albumin‐mediated systemic recycling, prolonged blood circulation, interstitial transport, and subsequent lymphatic drainage. By integrating time‐dependent biodistribution analysis, LC‐MS/MS quantification, and histological localization within LYVE1‐positive lymphatic sinus regions, we consistently observed preferential accumulation of PTX/BLZ945@Alb‐lip in residual tumors and TDLNs. Although these findings do not directly visualize the in vivo transport pathway from the circulation to lymphatic vessels and TDLNs, they are consistent with the physiological transport characteristics of albumin and suggest that lymphatic‐associated delivery contributes to the enhanced drug enrichment in TDLNs. Future studies employing real‐time lymphatic tracing techniques will be important for directly visualizing this transport pathway and further validating the proposed delivery mechanism.

Previous studies have explored MWA‐based immunotherapeutic strategies, including immune checkpoint blockade, myeloid modulation, and immune‐stimulatory nanomedicine, to overcome tumor recurrence and metastasis after local ablation [10, 39, 40]. These approaches share a common therapeutic objective with our study, namely overcoming tumor recurrence and metastasis after local ablation through immune modulation. However, most existing strategies have focused on the residual tumor microenvironment, whereas the role of TDLNs in post‐ablation immune regulation remains largely unexplored. Guided by scRNA‐seq analysis of post‐iMWA TDLNs, we identified an immunosuppressive macrophage niche characterized by expanded *Folr2^+^* and *Apoe^+^* macrophage subsets, elevated *Csf1r* expression, and enhanced CSF‐associated communication. Based on these findings, we developed a PTX/BLZ945@Alb‐lip‐based strategy to simultaneously remodel residual tumors and TDLNs, thereby enhancing systemic antitumor immunity, suppressing recurrence, and inducing durable immune memory.

Although albumin‐based PTX/BLZ945 nanoplatforms have previously been reported for macrophage modulation and tumor immunotherapy [41], the present albumin‐coated liposomal system differs from previous platforms in its material architecture, administration route, and delivery characteristics. Unlike previously reported locally administered stimulus‐responsive nanocarriers, PTX/BLZ945@Alb‐lip is designed for systemic delivery and exploits albumin‐associated biological transport properties to enhance drug accumulation in both residual tumors and TDLNs. Guided by scRNA‐seq analyses of post‐ablation TDLNs, we identified an immunosuppressive macrophage niche characterized by enhanced CSF1R‐associated signaling following iMWA and established a therapeutic strategy that coordinately remodels the immune microenvironments of residual tumors and TDLNs. By integrating distinct nanomaterial properties with mechanism‐guided immune modulation, this strategy provides a systemic approach for suppressing tumor recurrence and promoting durable antitumor immune memory after ablation.

A recent study demonstrated that PD1/PDL1 interactions within lymph nodes, rather than within tumors, correlated with prognosis in melanoma patients [18]. This clinical observation further supports the importance of immune regulation within TDLNs. Consistent with these findings, we observed marked infiltration of immunosuppressive macrophage subsets into TDLNs following iMWA.

The results from several clinical trials conducted on both melanoma and breast cancer patients have consistently shown that lymphadenectomy does not confer any overall survival benefit, even in cases where the sentinel lymph node is involved [42, 43]. It is conceivable that careful consideration should be given to the dissection of lymph nodes in circumstances where resection may potentially compromise the abscopal effect induced by systemic therapies such as immunotherapy. Our results from the TDLN resection study also emphasize the critical role of restoring TDLN immune function in enhancing antitumor immunity. These findings suggest that preservation of functional TDLNs may further enhance the efficacy of ablation‐associated immunotherapy. Therefore, therapeutic strategies that redirect immune responses within lymph nodes from tolerance to activation may offer a promising approach for enhancing antitumor immunotherapy.

Despite these encouraging findings, several limitations should be acknowledged. First, although BLZ945‐mediated pharmacological inhibition combined with scRNA‐seq analyses supports the involvement of CSF1R‐associated signaling in post‐ablation immune remodeling, further validation using myeloid‐specific *Csf1r* conditional knockout models or CSF1R‐blocking antibodies would provide more rigorous mechanistic evidence. Second, because post‐ablation TDLNs are rarely available in clinical practice, whether the CSF1R‐associated macrophage remodeling identified in our mouse model also occurs in patients remains to be further investigated. Although analyses of publicly available human datasets support the clinical relevance of the CSF1‐CSF1R axis, direct validation using post‐ablation patient TDLNs is still lacking. Third, although PTX/BLZ945@Alb‐lip demonstrated promising therapeutic efficacy and favorable biocompatibility in murine models, further optimization of drug dosage and administration schedules, together with pharmacokinetic and long‐term safety evaluations and validation in large‐animal models, will be required before clinical translation.

In summary, our findings provide robust evidence supporting the existence of an immunosuppressive microenvironment in TDLNs following iMWA, which is predominantly attributed to the increased infiltration of specialized macrophage subsets that express *Csf1r*. Accordingly, we have developed a highly potent albumin‐based nanosystem designed to deliver PTX and the CSF1R inhibitor BLZ945 to tumors and TDLNs for enhanced postablative immunotherapy. By utilizing multiple tumor models, we have demonstrated that the PTX/BLZ945@Alb‐lip strategy elicits robust antitumor immune responses, as evidenced by the augmented frequency of M1‐like macrophages and CD8^+^ T cell populations in both the lymph nodes and tumor site. This leads to a significant deceleration in local tumor progression following ablation treatment and confers protection against subsequent tumor rechallenge. Finally, the lipid and albumin components used to construct the nanosystem possess well‐established biocompatibility, and together with the encouraging therapeutic efficacy observed in this study, support the translational potential of this strategy. Safety investigations demonstrate that PTX/BLZ945@Alb‐lip does not induce any discernible impairment of liver and kidney function or pathological tissue changes during the studied duration. As a widely accepted modality for locoregional treatment in clinical practice, ablation therapies generally maintain the integrity of lymph nodes. The successful translation of this approach is expected to significantly influence the administration of anticancer immunotherapy in interventional oncology.

## 4 Experimental Section

### 4.1 Materials

Distearoyl phosphatidylcholine (DSPC), 1,2‐dioleoyl‐3‐trimethylammonium‐propane (DOTAP), 1,2‐distearoyl‐sn‐glycero‐3‐phosphoethanolamine‐N‐[amino(polyethylene glycol)2000] (DSPE‐PEG‐2000), Cy5.5‐DSPE‐PEG‐2000, and cholesterol were procured from Xi'an Ruixi Biological Technology Co., Ltd. Bovine serum albumin (BSA) was acquired from Proliant Biologicals. BLZ945 was provided by Shanghai Rhawn Chemical Technology Co., Ltd. Paclitaxel (PTX) was purchased from Shenyang Biological Pharmaceutical Co., Ltd. Dimethyl sulfoxide (DMSO) and anhydrous ethanol were obtained from Sinopharm Chemical Reagent Co., Ltd. The ELISA kit was sourced from Multi Sciences.

### 4.2 Formation of the PTX/BLZ945@Alb‐lip Lipoprotein Nanosystem

First, PTX/BLZ945@Lip was fabricated using a modified reverse evaporation method [44]. BLZ945 and PTX were dissolved in 10 mL of anhydrous ethanol containing DSPC, cholesterol, DOTAP, and DSPE‐PEG‐2000 at a fixed weight ratio of 10.18:3.28:1.00:3.16. The lipid film was formed by removing the anhydrous ethanol with a rotary evaporator at 50°C for 1 h. Five milliliters of PBS (0.01 m, pH 7.6) was added to the lipid film and mixed thoroughly. Subsequently, PTX/BLZ945@Lip was obtained through an extrusion process conducted with a mini‐extruder using 200 and 100 nm pore‐size membranes sequentially. For the preparation of the BSA‐coated cationic liposome, albumin was fully dissolved in 5 mL of phosphate buffer solution (pH 7.4). The resulting solution, with a concentration of 800 µg mL^−1^, was introduced into the prepared cationic liposome suspension and incubated at 37°C for 1 h. After sufficient binding of albumin to the cationic liposome via electrostatic interactions, centrifugation was performed at 12 000 rpm for 20 min to remove unbound albumin, resulting in the formation of PTX/BLZ945@Alb‐lip.

### 4.3 Characterization of PTX/BLZ945@Alb‐lip

The hydrodynamic particle size and zeta potential of PTX/BLZ945@Alb‐lip were determined using the Malvern Zetasizer Nanoseries (Nano ZS90). The morphology of PTX/BLZ945@Alb‐lip was evaluated by transmission electron microscopy (TEM) using the FEI TECNAI G2 12. The BSA content of PTX/BLZ945@Alb‐lip was characterized by sodium dodecyl sulfate‐polyacrylamide gel electrophoresis (SDS‐PAGE). The encapsulation of BLZ945 in PTX/BLZ945@Alb‐lip was confirmed using UV‐vis spectroscopy (Shimadzu UV‐3101 PC). The encapsulation efficiency and drug release profile of PTX in PTX/BLZ945@Alb‐lip were assessed by high‐performance liquid chromatography (HPLC) (Shimadzu LC‐20AD). To evaluate stability, PTX/BLZ945@Alb‐lip was stored at 4°C for 7 days, during which the particle size and polydispersity index (PDI) were monitored over time.

### 4.4 Cell Lines and Mice

The 4T1 cell line and the RAW264.7 cell line were originally obtained from the American Type Culture Collection (ATCC). All cells were cultured in the recommended medium and under the specified conditions. The cells were tested every two months for mycoplasma contamination. Female BALB/c mice, aged 6 to 8 weeks, were acquired from Beijing Vital River Laboratory Animal Technology Co., Ltd.

### 4.5 Macrophage Activation Assay

RAW264.7 cells were stimulated with IL‐4 (20 ng mL^−1^) and IL‐13 (10 ng mL^−1^). The supernatant from each well was collected and analyzed for cytokine secretion, including IL‐12, TNF‐α, TGF‐β, and IL‐10, using enzyme‐linked immunosorbent assay.

### 4.6 Enzyme‐Linked Immunosorbent Assay (ELISA)

The supernatant medium and serum were collected from cells and mice following various treatments. The levels of IFN‐γ (YOBIBIO, Catalog No. U96‐1475E), IL‐12 (Multi Sciences, Catalog No. EK212/3‐96), TNF‐α (Multi Sciences, Catalog No. EK282/4‐96), TGF‐β (Multi Sciences, Catalog No. EK981‐96), and IL‐10 (Multi Sciences, Catalog No. EK210/4‐96) in the supernatant and serum were quantified using ELISA kits according to the manufacturer's instructions.

### 4.7 Co‐Culture of IL‐4/IL‐13 Treated Macrophages with 4T1 Cells

In the co‐culture experiments, 4T1 cells were placed in the lower chamber of 0.4‐µm pore‐sized Transwell inserts (Corning) in 24‐well plates, while IL‐4/IL‐13 treated macrophages were added to the upper chamber and incubated for 12 h. The 4T1 cells were cultured independently and used in subsequent assays.

### 4.8 Cell Counting Kit‐8 (CCK‐8)

4T1 cells, RAW264.7 cells, and BMDCs were seeded into 96‐well plates at a density of 3 × 10^3^ cells per well. These cells were then subjected to various treatments for 24 h. Cell viability was assessed using the CCK‐8 method.

### 4.9 Confocal Laser Scanning Microscopy (CLSM)

Upon collection from the Transwell coculture chambers, 4T1 cells were seeded into CLSM‐specific culture dishes at a density of 10^5^ cells per dish and cultivated for 12 h. The 4T1 cells were then treated with PBS, PTX, BLZ945, DOX, GEM, PB, DB, and GB at a concentration of 100 µg mL^−1^ for 24 h. Subsequently, live and dead 4T1 cells were stained with Calcein‐AM/PI and analyzed using CLSM.

### 4.10 Tumor Models and Treatment Experiments

The BALB/c mice, aged 6 to 8 weeks, were inoculated with 1 × 10^6^ 4T1 cells or 1 × 10^6^ luc‐4T1 cells into the right mammary fat pad. When the tumors grew to approximately 8 mm in diameter (10 days after tumor implantation), the mice were randomly divided into different treatment groups. A post‐ablative tumor model was established using a microwave generator (ECO‐100E, Yigao Microwave Electric Institute, Nanjing, China). Following anesthesia in 4T1 tumor‐bearing mice, the iMWA antenna was percutaneously inserted under sterile conditions into the center of the tumor's long axis. The ablation duration was maintained between 1.0 and 1.5 min, with the ablation power set at 5 W. On day 3 post‐treatment, lymph nodes were harvested for single‐cell sequencing, immunofluorescence analysis, and flow cytometric analysis.

In the study of immunotherapy utilizing PTX/BLZ945@Alb‐lip in the 4T1 mouse tumor model, 4T1 mammary carcinoma cells were injected into the right mammary fat pad of BALB/c mice. Once the maximum tumor diameter reached approximately 8 mm (10 days post‐implantation), the post‐ablative tumor model was established as described previously. The mice were subsequently divided into five groups and administered different preparations via tail vein injections on days 1, 4, 7, and 10 after ablation: PBS, BLZ945/PTX, BLZ945@Alb‐lip, PTX@Alb‐lip, and PTX/BLZ945@Alb‐lip (with BLZ945 at 5 mg kg^−1^ and PTX at 10 mg kg^−1^). Bioluminescence imaging was conducted using an IVIS after 60 s of exposure to monitor residual tumor progression. Additionally, tumor size was measured every 2 days with calipers, and volume was estimated using the formula $(\mathrm{long}\;\mathrm{diameter}\;\times {\;\mathrm{short}\;\mathrm{diameter}}^{2})/2$. Mice were weighed, and survival curves were generated. Lymph nodes and tumors were excised for various analyses, including multiple immunohistochemistry assays, flow cytometry, H&E staining, and immunofluorescence staining. To evaluate the long‐term anticancer immune memory effect following PTX/BLZ945@Alb‐lip treatment, four cured mice that survived for 50 days were selected from the treatment group. Furthermore, four naive mice of the same sex and age were chosen as controls. 4T1 breast cancer cells were injected into the left (contralateral) mammary fat pads of both groups, and tumors were assessed using the same methodology as previously described. Finally, the spleens of the mice were collected and analyzed via flow cytometry.

### 4.11 Surgical Resection of TDLNs

The tumor model following ablation was established as described in the previous section. Mice were randomly allocated into four experimental groups: the control group (G1), the TDLN resection group (G2), the TDLN resection combined with PTX/BLZ945@Alb‐lip administration group (G3), and the PTX/BLZ945@Alb‐lip group (G4). For mice in groups G2 and G3, small incisions were made in the right groin region. The TDLNs were carefully identified and isolated using blunt forceps, followed by complete excision with sharp forceps. The incisions were then sutured to ensure proper wound closure.

On days 1, 4, 7, and 10 post‐treatment, the four groups received intravenous injections via the tail vein as follows: G1 and G2 received PBS, while G3 and G4 were administered PTX/BLZ945@Alb‐lip (BLZ945: 5 mg kg^−1^; PTX: 10 mg kg^−1^). Residual tumors were subsequently monitored and analyzed using the previously described methods.

### 4.12 In Vivo Biodistribution of PTX/BLZ945@Alb‐lip

Mice with incompletely ablated 4T1 mammary carcinomas were randomly assigned to two groups and injected via the tail vein with fluorescent Cy5.5‐labeled PTX/BLZ945@Alb‐lip and PTX/BLZ945@Lip. An IVIS was employed at various time points (0, 8, 12, 24, and 48 h) to observe and record fluorescence intensity in the lymph nodes and residual tumors. At 8 h, a separate subset of mice was sacrificed, and the lymph nodes, residual tumors, and major organs (heart, liver, spleen, lungs, and kidneys) were harvested for ex vivo fluorescence imaging to assess the distribution of the different delivery systems within lymph nodes, residual tumors, and major organs.

### 4.13 Single‐Cell RNA Sequencing (scRNA‐seq)

TDLNs from the control and iMWA groups were dissociated into single‐cell suspensions and subjected to scRNA‐seq using the Chromium Single Cell 3′ Library & Gel Bead Kit v3 (10x Genomics, Pleasanton, CA, USA). Libraries were sequenced on an Illumina NovaSeq 6000 platform in paired‐end 150‐bp (PE150) mode, generating approximately 100 Gb of sequencing data per sample, by OE Biotech Co., Ltd. (Shanghai, China).

Raw sequencing data were processed using the Cell Ranger pipeline and aligned to the mouse reference genome (mm10) to generate gene–barcode expression matrices. Cell Ranger initially identified 11 064 cells in the control group and 10 146 cells in the iMWA group. Quality control was performed using the Seurat package (R) as follows. Doublets were removed using DoubletFinder. Cells with fewer than 200 detected genes, fewer than 1000 UMIs, log10GenesPerUMI < 0.7, a mitochondrial UMI proportion > 10%, or a hemoglobin gene proportion > 5% were excluded, resulting in 9802 cells in the control group and 8992 cells in the iMWA group for downstream analyses.

Gene expression matrices were normalized using the LogNormalize method implemented in Seurat. Highly variable genes were identified and principal component analysis (PCA) was performed for dimensionality reduction. Cell clustering was conducted using the shared nearest neighbor (SNN) algorithm, and Uniform Manifold Approximation and Projection (UMAP) was used for visualization. Cell identities were assigned according to canonical marker genes identified by the FindAllMarkers function together with established immune‐cell markers. Differentially expressed genes (DEGs) were identified using the FindMarkers function with P < 0.05 and |log_2_ fold change| > 0.58 as significance thresholds. GO and KEGG enrichment analyses were subsequently performed using R.

To characterize macrophage functional programs, immunosuppressive signatures were quantified using the AddModuleScore function in Seurat. Cell–cell communication was inferred using the CellChat package (version 2.1.2), in which overexpressed ligand–receptor pairs were identified and communication probabilities were calculated and aggregated into signaling networks. Pathway enrichment analyses were conducted using Gene Set Enrichment Analysis (GSEA) and Gene Set Variation Analysis (GSVA) following standard workflows.

### 4.14 Liquid Chromatography–Tandem Mass Spectrometry (LC‐MS/MS)

PTX and BLZ945 concentrations in tumors and TDLNs were determined using a Thermo Scientific Vanquish UHPLC system coupled with a TSQ Altis Plus triple quadrupole mass spectrometer (Thermo Fisher Scientific, Waltham, MA, USA). Chromatographic separation was achieved on an Agilent ZORBAX Eclipse Plus C18 column (2.1 × 150 mm, 1.8 µm). Quantification was performed in multiple reaction monitoring (MRM) mode using Xcalibur 4.5 software, and drug concentrations were expressed as ng g^−^^1^ tissue.

### 4.15 Flow Cytometry

Small tissue samples were dissected from the lymph nodes, tumors, and spleens collected from mice and homogenized in staining buffer to generate single‐cell suspensions in the presence of digestive enzymes. The cells were subsequently stained with fluorescently labeled antibodies: CD3‐PE‐Cy7 (Thermo, Catalog No. 25‐0031‐82), CD8‐PerCP‐Cy5.5 (BioLegend, Catalog No. 100734), CD44‐PE (Thermo, Catalog No. 12‐0441‐82), CD62L‐APC (Thermo, Catalog No. 17‐0621‐82), CD4‐FITC (Thermo, Catalog No. 11‐0042‐85), CD45‐BV510 (BioLegend, Catalog No. 103138), CD11b‐BUV395 (Thermo, Catalog No. 363‐0112‐82), Ly6C‐PerCP‐Cy5.5 (BioLegend, Catalog No. 128012), Ly6G‐eF450 (Thermo, Catalog No. 48‐9668‐82), F4/80‐APC (Thermo, Catalog No. 17‐4801‐82), CD86‐FITC (Thermo, Catalog No. 11‐0862‐85), CD206‐PE (BioLegend, Catalog No. 141706), CD25‐APC (Thermo, Catalog No. 17‐0251‐82), and Foxp3‐PE (Thermo, Catalog No. 12‐5773‐82), following the manufacturer's instructions. All antibodies were diluted 100‐fold. A Fortessa X20 flow cytometer (Becton, Dickinson and Company) was utilized to assess the stained cells, and the analysis was performed using FlowJo software (version 10.9.0, TreeStar). Percentages are based on the data obtained from flow cytometry analysis.

### 4.16 Multiplex Immunofluorescence (mIF)

Formalin‐fixed paraffin‐embedded (FFPE) lymph node and tumor tissues were sectioned and subjected to multiplex immunofluorescence staining according to the manufacturer's instructions. The following primary antibodies were used: Ki‐67 (Starter, Catalog No. S0B2332), CD206 (Cell Signaling Technology, Catalog No. 24595), CD8 (Starter, Catalog No. S0B0034), and FoxP3 (Abcam, Catalog No. ab215206). Stained slides were scanned using a Pannoramic MIDI digital slide scanner (3DHISTECH).

### 4.17 Immunohistochemistry (IHC)

FFPE lymph node and tumor sections were stained with a primary antibody against CSF1R (Proteintech, Catalog No. 25949‐1‐AP) according to the manufacturer's instructions. Stained slides were scanned using a Pannoramic MIDI digital slide scanner (3DHISTECH).

### 4.18 Immunofluorescence Staining

Tumor‐draining lymph nodes were collected and embedded in optimal cutting temperature (OCT) compound. Frozen sections were cryosectioned and stained with LYVE‐1 (Cell Signaling Technology, Catalog No. 98947) according to the manufacturer's instructions. Nuclei were counterstained with DAPI, and the Cy5 fluorescence of liposomes was detected directly. Images were acquired using a Pannoramic MIDI digital slide scanner (3DHISTECH).

### 4.19 Statistical Analysis

All data are presented as mean ± standard deviation (SD). Statistical analyses were performed using GraphPad Prism 8. For comparisons between two groups, an unpaired Student's *t*‐test was used. For comparisons among multiple groups, one‐way analysis of variance (ANOVA) followed by Tukey's multiple comparisons test was applied. Survival curves were analyzed using the log‐rank (Mantel–Cox) test. A *p* value < 0.05 was considered statistically significant unless otherwise indicated.

## Author Contributions

**Xuexia Shan**: Writing – original draft, Investigation, Data curation. **Yuting Shen**: Investigation. **Xiaodong Hou**: Data curation. **Fengshan Jin**: Data curation. **Shaoyue Li**: Data curation. **Weichen Xu**: Methodology. **Weiwei Ren**: Methodology. **Xiaowan Bo**: Visualization. **Weifan Hao**: Visualization. **Qiuyan Li**: Formal analysis. **Shuo Liu**: Formal analysis. **Chengzhong Peng**: Methodology. **Liping Sun**: Supervision. **Huixiong Xu**: Supervision. **Wenwen Yue**: Writing – review & editing, Supervision, Project administration, Funding acquisition.

## Ethics statement

All mice were maintained in accordance with the policies set forth by the National Ministry of Health. All mouse experiments conducted throughout the study were approved by the Laboratory Animal Center of Shanghai Tenth People's Hospital (SHDSYY‐2024‐6095‐2).

## Conflicts of Interest

The authors declare no conflicts of interest.

## Supporting information




**Supporting File 1**: advs78058‐sup‐0001‐SuppMat.docx.

**Supporting File 2**: advs78058‐sup‐0002‐SuppMat.docx.

**Supporting File 3**: advs78058‐sup‐0003‐TableS1.xls.

## Data Availability

All data are in the main text or the supplementary materials. All raw data generated in this study are available upon request from the corresponding author.
