# Supplementary material for: Targeting Macrophage‐Mediated Lymphatic Immunosuppression to Prevent Tumor Recurrence After Microwave Ablation
Source: Adv Sci (Weinh). 2026 Sep 28:e78058. Online ahead of print. doi: 10.1002/advs.78058 (PMC13620260; doi:10.1002/advs.78058)
Supplement: Supplementary file 1 [file ADVS-9999-e78058-s001.docx]

Supporting Information

**Targeting Macrophage-Mediated Lymphatic Immunosuppression to Prevent Tumor Recurrence after Microwave Ablation**

*Xuexia Shan, Yuting Shen, Xiaodong Hou, Fengshan Jin, Shaoyue Li, Weichen Xu, Weiwei Ren, Xiaowan Bo, Qiuyan Li, Shuo Liu, Weifan Hao, Chengzhong Peng, Huixiong Xu* , Liping Sun*, Wenwen Yue**

Correspondence to: xuhuixiong@126.com; sunliping_s@126.com; yuewen0902@163.com

**Figure S1.** Heatmap depicting the interaction strength among various immune cell types in the iMWA group. The vertical axis represents ligand-expressing cell types and the horizontal axis represents receptor-expressing cell types. The color gradient indicates the relative interaction strength between cell types. The bar plots on the top and right margins represent the summed incoming and outgoing signaling strengths for each cell population.

**Figure S2.** The heatmap depicting the ranking of the marker genes for each subpopulation of macrophages.

**Figure S3.** Dot plot showing the expression levels of functional gene sets across macrophage subsets.

**Figure S4.** GSVA analysis of GO analysis of macrophage subsets in TDLNs.

**Figure S5.** (A) The UMAP plot representing the major subpopulations of T cells. (B) The heatmap depicting the marker genes ranked in the top 10 for each subpopulation of T cells.

**Figure S6**. Network visualization highlighting signaling interactions between macrophage and T cell subtypes under the CSF pathway in the control group. Edge thickness is proportional to the interaction strength, and edge color corresponds to the ligand-producing (source) cell.

**Figure S7**. Clinical relevance of the CSF1-CSF1R axis in human breast cancer. Human breast cancer scRNA-seq datasets (GSE167036, GSE225600, and GSE180286) and TCGA-BRCA bulk RNA-seq were analyzed. All human samples were obtained from patients without prior thermal ablation. (A) UMAP of GSE167036 macrophages showing macrophage subclusters, *CSF1R* expression, and mouse-derived *Folr2⁺* and *Apoe⁺* macrophage signatures. (B) Module scores of the mouse-derived *Folr2⁺* and *Apoe⁺* macrophage signatures across breast cancer molecular subtypes in GSE180286. (C) Dot plot showing the expression of *CSF1*, *CSF1R*, and representative macrophage-associated genes across major cell populations in GSE167036. (D) Correlation between representative CSF1-CSF1R axis genes and macrophage infiltration scores in TCGA-BRCA. (E) Correlation between *CSF1R* expression and macrophage infiltration scores in TCGA-BRCA. (F) Heatmap showing the expression of CSF1-CSF1R signaling components and representative macrophage-associated genes in paired primary tumors (PT) and lymph nodes (LN) from GSE225600. (G) Violin plots showing representative immunoregulatory macrophage-associated genes in CSF1R-high and CSF1R-low macrophages from GSE167036.

**Figure S8.** (A-C) ELISA quantification of IL-10 (A), IL-12 (B), and TGF-β (C) levels in the culture medium of RAW264.7-derived macrophages following different drug treatments. PTX (5 μm), BLZ945 (5 μm), DOX (5 μm), GEM (5 μm), PB (PTX 2.5 μm + BLZ945 2.5 μm), DB (DOX 2.5 μm + BLZ945 2.5 μm), and GB (GEM 2.5 μm + BLZ945 2.5 μm). Data are presented as mean ± SD (*n* = 3). Statistical significance was determined using one-way ANOVA followed by Tukey’s multiple comparisons test.

**Figure S9.** Cell viability of 4T1 breast cancer cells co-cultured with IL-4/IL-13–stimulated RAW264.7 macrophages was determined using the Cell Counting Kit-8 (CCK-8) assay. Data are presented as mean ± SD (*n* = 3). Statistical significance was determined using one-way ANOVA followed by Tukey’s multiple comparisons test.

**Figure S10.** (A, B) ELISA analysis showing the levels of TNF-α (A) and IL-10 (B) in the culture medium of RAW264.7-derived macrophages treated with PTX (1, 5, and 10 μm), BLZ945 (1, 5, and 10 μm), or their combinations. Data are presented as mean ± SD *(n* = 3).

**Figure S11.** The zeta potential of Lip, Alb-lip, BLZ945@Alb-lip, and PTX/BLZ945@Alb-lip. Data are presented as mean ± SD *(n* = 3).

**Figure S12.** SDS-polyacrylamide gel electrophoresis analysis of Lip, Alb, Alb-lip, and PTX/BLZ945@Alb-lip.

**Figure S13.** The UV-vis absorbance spectra of Lip, Alb-lip, BLZ945, BLZ945@Alb-lip, and PTX/BLZ945@Alb-lip.

**Figure S14.** The successful co-loading of PTX into PTX/BLZ945@Alb-lip was evidenced by the high-performance liquid chromatography (HPLC) results of PTX, PTX@Alb-lip and PTX/BLZ945@Alb-lip.

**Figure S15.** The size and PDI of PTX/BLZ945@Alb-lip in PBS with 10% serum at 4 °C were monitored over time.

**Figure S16.** (A, B) The drug-loading efficiency (A) and capacity (B) of BLZ945 and PTX within the engineering lipoprotein nanosystem were determined by means of UV-vis absorbance spectra and HPLC at various drug concentrations.

**Figure S17.** (A–C) ELISA analysis of IL-10 (A), IL-12 (B), and TGF-β (C) levels in the culture supernatant of IL-4/IL-13–induced RAW264.7 macrophages following different treatments. Data are expressed as mean ± SD (*n* = 3). Statistical significance was determined using one-way ANOVA followed by Tukey’s multiple comparisons test.

**Figure S18.** The survival rate of 4T1 breast cancer cells treated singly with diverse treatments was ascertained by means of CCK-8. P/B@A-l, PTX/BLZ945@Alb-lip. Data are presented as mean ± SD (*n* = 3). Statistical significance was determined using one-way ANOVA followed by Tukey’s multiple comparisons test.

**Figure S19.** Cytocompatibility of PTX/BLZ945@Alb-lip toward immune cells. (A, B) Cell viability of RAW264.7 macrophages (A) and mouse bone marrow-derived dendritic cells (BMDCs) (B) after treatment with PBS, PTX, BLZ945, PB, or PTX/BLZ945@Alb-lip at the concentrations used in the in vitro functional assays, as determined by the CCK-8 assay. BMDC, bone marrow-derived dendritic cell. Data are presented as mean ± SD (*n* = 3).

**Figure S20.** (A, B) Quantitative biodistribution of PTX (A) and BLZ945 (B) in tumors and TDLNs 8 h after intravenous administration. Drug concentrations were quantified by liquid chromatography–tandem mass spectrometry (LC-MS/MS). P/B@L, PTX/BLZ945@Lip; P/B@Alb-lip, PTX/BLZ945@Alb-lip. Data are presented as mean ± SD (*n* = 3). Statistical significance was analyzed using an unpaired two-tailed Student's *t*-test.

**Figure S21.** Representative immunofluorescence images of Cy5-labeled PTX/BLZ945@Lip and PTX/BLZ945@Alb-lip (red) in TDLNs. LYVE-1-positive lymphatic sinus structures are shown in green and nuclei are counterstained with DAPI (blue). Representative higher-magnification images (70×) are shown on the right. P/B@L, PTX/BLZ945@Lip; P/B@Alb-lip, PTX/BLZ945@Alb-lip. Scale bars = 500 μm and 20 μm.

**Figure S22.** (A, B) Representative flow cytometry (A) and quantitative analysis (B) of M1-TAMs (CD86^+^CD11b^+^F4/80^+^CD45^+^) within lymph nodes. G1, Control; G2, BLZ945/PTX; G3, BLZ945@Alb-lip; G4, PTX@Alb-lip; G5, PTX/BLZ945@Alb-lip. Data are presented as mean ± SD (*n* = 3). Statistical significance was determined using one-way ANOVA followed by Tukey’s multiple comparisons test.

**Figure S23.** (A, B) Representative flow cytometry (A) and quantitative analysis (B) of M2-TAMs (CD206^+^CD11b^+^F4/80^+^CD45^+^) within tumors. G1, Control; G2, BLZ945/PTX; G3, BLZ945@Alb-lip; G4, PTX@Alb-lip; G5, PTX/BLZ945@Alb-lip. Data are presented as mean ± SD (*n* = 3). Statistical significance was determined using one-way ANOVA followed by Tukey’s multiple comparisons test.

**Figure S24.** (A, B) Representative flow cytometry (A) and quantitative analysis (B) of M1-TAMs (CD86^+^CD11b^+^F4/80^+^CD45^+^) within tumors. G1, Control; G2, BLZ945/PTX; G3, BLZ945@Alb-lip; G4, PTX@Alb-lip; G5, PTX/BLZ945@Alb-lip. Data are presented as mean ± SD (*n* = 3). Statistical significance was determined using one-way ANOVA followed by Tukey’s multiple comparisons test.

**Figure S25.** (A, B) Representative flow cytometry (A) and quantitative analysis (B) of CD8^+^ T cells (CD8^+^CD3^+^CD45^+^) within tumors. G1, Control; G2, BLZ945/PTX; G3, BLZ945@Alb-lip; G4, PTX@Alb-lip; G5, PTX/BLZ945@Alb-lip. Data are presented as mean ± SD (*n* = 3). Statistical significance was determined using one-way ANOVA followed by Tukey’s multiple comparisons test.

**Figure S26.** (A, B) Representative flow cytometry (A) and quantitative analysis (B) of Tregs (CD25^+^Foxp3^+^CD4^+^CD3^+^CD45^+^) within tumors. G1, Control; G2, BLZ945/PTX; G3, BLZ945@Alb-lip; G4, PTX@Alb-lip; G5, PTX/BLZ945@Alb-lip. Data are presented as mean ± SD (*n* = 3). Statistical significance was determined using one-way ANOVA followed by Tukey’s multiple comparisons test.

**Figure S27.** Quantification of theM1/M2 ratio in tumors by flow cytometry. G1, Control; G2, BLZ945/PTX; G3, BLZ945@Alb-lip; G4, PTX@Alb-lip; G5, PTX/BLZ945@Alb-lip. Data are presented as mean ± SD (*n* = 3). Statistical significance was determined using one-way ANOVA followed by Tukey’s multiple comparisons test.

**Figure S28.** Quantification of the CD8⁺ T cell/Treg ratio in tumors by flow cytometry. G1, Control; G2, BLZ945/PTX; G3, BLZ945@Alb-lip; G4, PTX@Alb-lip; G5, PTX/BLZ945@Alb-lip. Data are presented as mean ± SD (*n* = 3). Statistical significance was determined using one-way ANOVA followed by Tukey’s multiple comparisons test.

**Figure S29.** Representative multiplex immunofluorescence images of the tumors (scale bar = 50 μm).

**Figure S30.** Representative immunohistochemical images of CSF1R (yellow) in the tumors (scale bar = 50 μm).

**Figure S31.** Whole-section images of TDLNs corresponding to the immunostaining analyses in Figure 5K, L. (A) Whole-section overview of multiplex immunofluorescence staining. The white box indicates the region shown at higher magnification in Figure 5K. (B) Whole-section overview of CSF1R immunohistochemical staining. The black boxes indicate the regions shown at higher magnification in Figure 5L (scale bar = 500 μm).

**Figure S32.** PTX/BLZ945@Alb-lip exhibits intrinsic antitumor activity and enhanced efficacy in the iMWA-associated setting. (A) Individual tumor growth curves in mice receiving the indicated treatments. (B) Mean tumor growth curves of the four treatment groups. (C) Representative images of excised tumors collected at the experimental endpoint. (D) Quantification of terminal tumor weights. (E) Representative flow cytometry plots and quantitative analysis of M2-TAMs (CD206⁺CD11b⁺F4/80⁺CD45⁺) within tumors. (F) Representative flow cytometry plots and quantitative analysis of M1-TAMs (CD86⁺CD11b⁺F4/80⁺CD45⁺) within tumors. (G) Representative flow cytometry plots and quantitative analysis of CD8⁺ T cells (CD8⁺CD3⁺CD45⁺) within tumors. (H) Representative flow cytometry plots and quantitative analysis of Tregs (Foxp3⁺CD25⁺CD4⁺CD3⁺CD45⁺) within tumors. (I) Quantification of the M1/M2 macrophage ratio in tumors. (J) Quantification of the CD8⁺ T cell/Treg ratio in tumors. (K, L) Serum levels of IFN-γ (K) and TNF-α (L) measured by ELISA. G1, PBS; G2, PTX/BLZ945@Alb-lip; G3, iMWA + PBS; G4, iMWA + PTX/BLZ945@Alb-lip. G1 and G2 represent groups without prior iMWA, whereas G3 and G4 represent groups receiving iMWA before treatment. Data are presented as mean ± SD. *n* = 10 mice per group in (A, B); *n* = 6 mice per group in (C, D); *n* = 3 mice per group in (E–J); *n* = 4 mice per group in (K, L). Statistical significance was determined using one-way ANOVA followed by Tukey’s multiple comparisons test.

**Figure S33.** PTX/BLZ945@Alb-lip remodels the immunosuppressive immune microenvironment of TDLNs following iMWA. (A) Representative flow cytometry plots and quantitative analysis of M2-TAMs (CD206⁺CD11b⁺F4/80⁺CD45⁺) in TDLNs. (B) Representative flow cytometry plots and quantitative analysis of M1-TAMs (CD86⁺CD11b⁺F4/80⁺CD45⁺) in TDLNs. (C) Representative flow cytometry plots and quantitative analysis of CD8⁺ T cells (CD8⁺CD3⁺CD45⁺) in TDLNs. (D) Representative flow cytometry plots and quantitative analysis of Tregs (Foxp3⁺CD25⁺CD4⁺CD3⁺CD45⁺) in TDLNs. (E) Quantification of the M1/M2 macrophage ratio in TDLNs. (F) Quantification of the CD8⁺ T cell/Treg ratio in TDLNs. G1, PBS; G2, PTX/BLZ945@Alb-lip; G3, iMWA + PBS; G4, iMWA + PTX/BLZ945@Alb-lip. G1 and G2 represent groups without prior iMWA, whereas G3 and G4 represent groups receiving iMWA before treatment. Data are presented as mean ± SD (*n* = 3). Statistical significance was determined using one-way ANOVA followed by Tukey’s multiple-comparisons test.

**Figure S34.** The relative levels of alanine aminotransferase (ALT), aspartate aminotransferase (AST), alkaline phosphatase (ALP), blood urea nitrogen (BUN), and creatinine (Crea) in the serum subjected to various treatments. G1, Control; G2, BLZ945/PTX; G3, BLZ945@Alb-lip; G4, PTX@Alb-lip; G5, PTX/BLZ945@Alb-lip. Data are presented as mean ± SD (*n* = 3).

**Figure S35.** H&E-stained images of major organs (heart, liver, spleen, lung, and kidney) from mice subjected to various treatments (scale bar = 100 μm).

**Figure S36.** (A, B) Representative flow cytometry (A) and quantitative analysis (B) of M2-TAMs (CD206^+^CD11b^+^F4/80^+^CD45^+^) within tumors. G1, Control; G2, TDLN resection; G3, TDLN res+PTX/BLZ945@Alb-lip; G4, PTX/BLZ945@Alb-lip. Data are presented as mean ± SD (*n* = 3). Statistical significance was determined using one-way ANOVA followed by Tukey’s multiple comparisons test.

**Figure S37.** (A, B) Representative flow cytometry (A) and quantitative analysis (B) of Tregs (CD25^+^Foxp3^+^CD4^+^CD3^+^CD45^+^) within tumors. G1, Control; G2, TDLN resection; G3, TDLN res+PTX/BLZ945@Alb-lip; G4, PTX/BLZ945@Alb-lip. Data are presented as mean ± SD (*n* = 3). Statistical significance was determined using one-way ANOVA followed by Tukey’s multiple comparisons test.

**Figure S38.** The gating strategies used to determine the percentage of TAMs among CD45^+^ immune cells are shown in Figures 5A, 7H, S22A, S23A, S24A, S32E, S32F, S33A, S33B, and S36A.

**Figure S39.** The gating strategies used to determine the percentage of T cell subsets among CD45^+^ immune cells are shown in Figures 5C, 5E, 7I, S25A, S26A, S32G, S32H, S33C, S33D, and S37A.

**Figure S40.** The percentages of the Tcm and Tem subpopulations of CD8^+^ and CD4^+^ T cells in the spleen were determined through a gating strategy as presented in Figure 6G. Tem, effector memory T cells; Tcm, central memory T cells.
